# Co-receptor tropism prediction among 1045 Indian HIV-1 subtype C sequences: Therapeutic implications for India

**DOI:** 10.1186/1742-6405-7-24

**Published:** 2010-07-21

**Authors:** Ujjwal Neogi, Sreenivasa B Prarthana, George D'Souza, Ayesha DeCosta, Vijesh S Kuttiatt, Udaykumar Ranga, Anita Shet

**Affiliations:** 1 Department of Microbiology, St. John's Medical College and Hospital, St. John's National Academy of Health Sciences, Sarjapur Road, Bangalore-560034, India; 2 Infectious Diseases Clinic, St. John's Medical College and Hospital, St. John's National Academy of Health Sciences, Sarjapur Road, Bangalore-560034, India; 3 Department of Pediatrics, St. John's Medical College and Hospital, St. John's National Academy of Health Sciences, Sarjapur Road, Bangalore-560034, India; 4 Division of Global Health, Nobels Väg 9, Karolinska Institutet, 171 77, Stockholm, Sweden; 5 Molecular Biology and Genetics Unit, Jawaharlal Nehru Centre for Advanced Scientific Research, Jakkur P.O., Bangalore-560064, India

## Abstract

****Background**:**

Understanding co-receptor tropism of HIV-1 strains circulating in India will provide key analytical leverage for assessing the potential usefulness of newer antiretroviral drugs such as chemokine co-receptor antagonists among Indian HIV-infected populations. The objective of this study was to determine using *in silico *methods, HIV-1 tropism among a large number of Indian isolates both from primary clinical isolates as well as from database-derived sequences.

**Results:**

R5-tropism was seen in 96.8% of a total of 1045 HIV-1 subtype C Indian sequences. Co-receptor prediction of 15 primary clinical isolates detected two X4-tropic strains using the C-PSSM matrix. R5-tropic HIV-1 subtype C V3 sequences were conserved to a greater extent than X4-tropic strains. X4-tropic strains were obtained from subjects who had a significantly longer time since HIV diagnosis (96.5 months) compared to R5-tropic strains (20.5 months).

**Conclusions:**

High prevalence of R5 tropism and greater homogeneity of the V3 sequence among HIV-1 subtype C strains in India suggests the potential benefit of CCR5 antagonists as a therapeutic option in India.

## Background

After the discovery of the CD4 molecule as the major cellular receptor for HIV entry [1,2], multiple studies suggested the presence of a secondary cellular receptor for HIV entry into the human CD4 cell [3,4]. These co-receptors, particularly the chemokine receptors CCR5 and CXCR4, have been the subject of much research attempting to elucidate viral entry mechanisms, disease progression, antiretroviral therapy and vaccine development. Based on co-receptor usage, viral strains are classified into R5-tropic (those that use CCR5 for cellular entry), X4-tropic (those that use CXCR4) and dual tropic strains (those that use both co-receptors) [5]. Co-receptor tropism of individual viral strains can be delineated using reporter cells expressing different coreceptors; however such cell-based assays are labor-intensive, expensive and not appropriate for high throughput screening [6]. As an alternative, *in silico *strategies using computer simulation and bioinformatics have been developed to predict viral co-receptor usage from *env *gene sequence information [5-7]. Of late, the *in silico *approaches have been gaining popularity given the simplicity of this strategy and the fact that *env *sequences are increasingly becoming available globally.

The simplest method used for delineating HIV-1 tropism is known as the 'charge rule' [8], which relies on the charge of amino acids at positions 11 or 25 within the V3 loop when aligned against a consensus. Presence of positively charged amino acids (i.e. arginine, lysine, or histidine) in these positions typically is indicative of X4-tropism, while presence of other amino acid residues is associated with R5 phenotype [9]. Currently a number of tools are available online to predict the co-receptor usage on the basis of the V3 sequence. Such tools include among others, (i) Geno2Pheno which predicts whether the corresponding virus is capable of using CXCR4 as a co-receptor (R5/X4 or X4 variants) or not (R5 variants) [10], (ii) the distant segments (ds)Kernel which include relative positional information of segments in a string of symbols which detects R5-, X4- and R5X4-tropic strains[11], and (iii) WebPSSM using CPSSM, a genotypic predictor based on position-specific scoring matrices (PSSM) which detects R5- or X4- tropic strains specially designed and validated for HIV-1 subtype C [12]. Dual-tropic strains in C-PSSM are grouped with the X4- data set [12]. Till date, molecular epidemiological information from India has indicated that > 96% HIV-1 circulating strains are HIV-1 subtype C (Geographic search interface, Los Alamos database, accessed on February 2010) [13]. While X4-tropic HIV-1 subtype C strains have been widely reported from Africa [14-16], the presence of CXCR4 as a co-receptor to facilitate entry into the host cell is uncommon among Indian subtype C strains [17,18]. R5-tropic viruses constitute by far the predominant strains in India although recent reports indicate the occasional presence of HIV-1 subtype C X4-tropic strains [19-21].

We aimed to characterize co-receptor tropism of HIV-1 subtype C strains isolated from a clinical cohort in southern India, using three different online bioinformatics tools. Furthermore, we aimed to validate this strategy and expand our understanding of co-receptor tropism preference among Indian strains by extending this analysis to a total of 1030 V3 sequences of Indian origin available at Los Alamos databank.

## Methods

### Study population and sample collection

A single peripheral blood sample was collected from 15 ART-naïve patients (10 males, 5 females) attending the Infectious Disease Clinic at St. John's Medical Hospital, Bangalore, between 1 and 30 November 2009. Patient characteristics are described in Table 1. Routine CD4 count was performed using a dual-platform flow cytometer (FACSCalibur, BD, USA). Genomic DNA from whole blood was extracted using a commercial kit (QIAamp Blood DNA kit, Qiagen, Germany).

**Table 1:** Patient demographic details and predicted HIV-1 subtype C co-receptor tropism

Patient demographic details						HIV-1 Co-receptor Tropism				
**No**	**Patient ID**	**Age**	**Sex**	**Time since sero-diagnosis (months)**	**CD4 Count (cells/mm^3^)**	**Disease Stage**	**WebPSSM (C-PSSM)**		**Geno2pheno**	**(ds)Kernel**
									
							**Score**	**Tropism**		

1.	SJNAHS01	33	M	43	223	2^nd^	-29.39	R5	R5	R5

2.	SJNAHS02	53	F	4	247	2^nd^	-29.97	R5	R5	R5

3.	SJNAHS03	30	M	46	173	2^nd^	-23.28	R5	R5	R5

4.	SJNAHS04	37	M	63	538	1^st^	-16.09	X4	R5	R5

5.	SJNAHS06	36	F	1	122	3^rd^	-23.32	R5	R5	R5

6.	SJNAHS07	26	F	1	384	1^st^	-29.97	R5	R5	R5

7.	SJNAHS09	38	F	1	356	3^rd^	-25.74	R5	R5	R5

8.	SJNAHS10	38	M	105	438	3^rd^	-25.74	R5	R5	R5

9.	SJNAHS11	26	M	15	594	1^st^	-29.39	R5	R5	R5

10.	SJNAHS12	38	M	22	59	1^st^	-25.74	R5	R5	R5

11.	SJNAHS13	36	M	130	77	1^st^	-19.35	X4	X4	R5

12.	SJNAHS16	39	M	1	13	4^th^	-29.08	R5	R5	R5

13.	SJNAHS17	36	F	1	75	1^st^	-29.05	R5	R5	R5

14.	SJNAHS18	60	M	1	99	4^th^	-22.37	R5	R5	R5

15.	SJNAHS19	40	M	2	200	1^st^	-22.37	R5	R5	R5

Patient demographic characteristics and predicted co-receptor tropism in the South Indian cohort. CD4 count represents the value at the time of study. Co-receptor tropism was detected using C-PSSM, Geno2pheno and (ds)Kernel method. In C-PSSM a score of above -21.64 was considered predictive of X4-tropism. Disease stage according to WHO classification has been mentioned.

### Polymerase chain reaction and sequencing

The *env *gene portion encoding the V3-V5 region was amplified by nested polymerase chain reaction (PCR) from whole blood genomic DNA using iNtRON Taq Polymerase (Intron Biotech, South Korea). Primers were designed based on a consensus Indian sequence and modified from previously published reports [22,23]. The first round of PCR was carried out with a forward primer, FP1: 5-CACCGGCTTAGGCATCTCCTATGGCAGGAAGAA-3 and reverse primer RP1: 5-TAACCCTTCCAGGTACCCCCTTTTCTTTTA-3. The nested PCR was carried out with the forward primer, FP2: 5'-tgtaaaacgacggccagtCTGTTAAATGGCAGTCTAGC and reverse primer, RP2: 5'-caggaaacagctatgaccCACTTCTCCAATTGTCCCTCA. Primers FP2 and RP2 contain the M13 universal primer sequence (lower case), which was used for population based sequencing.

### Subtyping

HIV subtyping was carried out using three different tools i.e. REGA subtyping tools v2.0 http://www.bioafrica.net/rega-genotype/html/subtypinghiv.html[24], NCBI Viral Genotyping tools http://www.ncbi.nlm.nih.gov/projects/genotyping/formpage.cgi[25] and RIP 3.0 http://www.hiv.lanl.gov/content/sequence/RIP/RIP.html[26].

### Indian V3 sequence and co-receptor prediction

Sequences from our primary clinical isolates (n = 15) were pooled with 1030 Indian V3 sequences (additional file 1; accession numbers and sequence information) available from the Los Alamos database accessed on 7 Feb 2010. All available HIV-1 subtype C V3 sequences (*n *= 1114) were downloaded. Sequences containing a premature stop codon (*n *= 84) were excluded from the study. In all, 1045 sequences were analyzed *in silico *for co-receptor tropism using three different tools; (i) C-PSSM http://indra.mullins.microbiol.washington.edu/webpssm/, (ii) Geno2pheno [co-receptor] http://coreceptor.bioinf.mpi-inf.mpg.de/ and (iii) (ds)Kernel http://genome.ulaval.ca/hiv-dskernel/. To compare V3 characteristics of the R5 and X4 tropic strains, the consensus sequences of R5 and X4 tropic strains detected in C-PSSM were obtained using WebLogo http://weblogo.berkeley.edu/logo.cgi and consensus maker tool present in Los Alamos database http://www.hiv.lanl.gov/content/sequence/CONSENSUS/consensus.html. The mutation patterns of the cohort V3 loop sequences were compared with the consensus HIV-1 subtype C sequence and presented as additional file 2.

### Ethical aspects

This study was approved by the Institutional Ethical Review Board of St. John's Medical College and Hospital. Written informed consent was obtained from each participant prior to sample collection.

## Results

### Subtyping

All the 15 clinical isolates were detected as HIV-1 Subtype C; using three different subtyping tools, REGA subtyping tools v 2, NCBI Viral Genotyping tools and RIP 3.

### Co-receptor tropism

**Figure 1:**
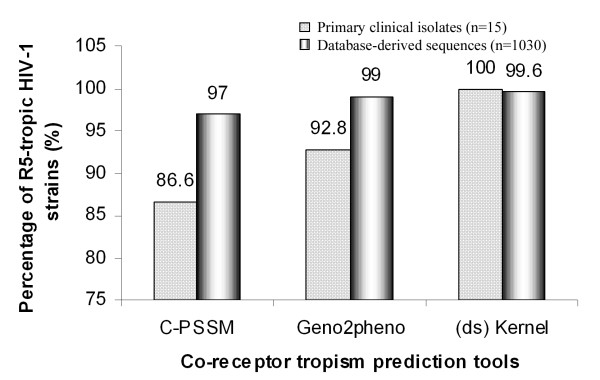
***In silico *viral tropism analysis**. Prediction of the viral co-receptor tropism using three different online tools C-PSSM, Geno2Pheno and (ds)Kernal. The analysis is applied to 15 primary viral isolates and a total 1030 V3 loop sequences derived from the Los Alamos database.

Table 1 summarizes the predicted co-receptor tropism of each of the individual viral strains derived from our clinical cohort. While all the three tools predicted 13 out of the 15 viruses to be R5-tropic, there was disagreement among them with respect to two viral strains. The viral strain SJNAHS04 was predicted to be an X4 virus by C-PSSM while the other two tools characterized it to be a R5 virus. Similarly, the strain SJNAHS13 was predicted to be an R5 virus by (ds)Kernel while the other two tools found it to be an X4 virus. To see how reliable the three bioinformatics tools were in predicting co-receptor tropism of subtype C strains, we applied each of these tools to 1030 Indian *env *V3 sequences available at the Los Alamos database. This analysis confirmed a high magnitude of R5-tropism in the Indian *env *sequences predicted by all the three tools, 97%, 99% and 99.6% of R5-tropic sequences by CPSSM, Geno2Pheno and (ds) Kernel, respectively (Figure 1). As multiple sequences may be derived from the same patient, a second analysis was done after eliminating sequences that were derived from the same individual, and the result was similar to that obtained when all included sequences were analyzed.

The score in C-PSSM used for coreceptor tropism prediction was previously validated using sequences of the known syncytium inducing (SI) phenotypes on the MT2 cell line [12]. C-PSSM score for SJNAHS04 (known history of HIV positivity > 5 years) was -16.09 and SJNAHS13 (known history of HIV positivity > 10 years) was -19.35; both were well above the prediction cutoff of -21.64 for X4-tropic viruses [12]. The remaining isolates characterized to be R5-tropic were obtained from subjects with a recent history of HIV diagnosis within the past 5 years. The mean duration since detection of HIV-1 infection was longer for X4-tropic strains compared to R5-tropic strains (96.5 months and 20.5 months respectively), although only 2 X4-tropic strains were available for this analysis.

### Sequence characteristics of Indian V3 sequence

**Figure 2:**
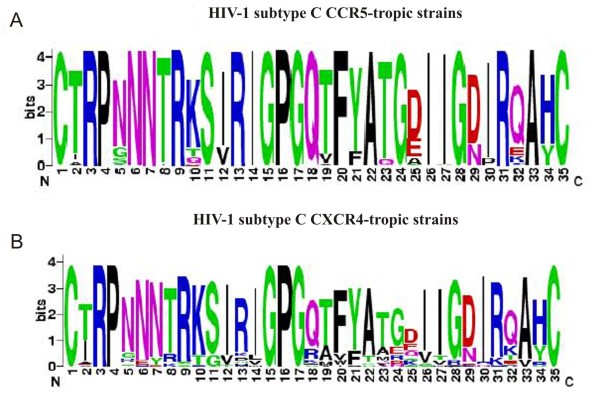
**Consensus sequence logos of the Indian V3 sequences**. **A**. HIV-1 subtype C CCR5-tropic strains (n = 1012), **B**. Subtype C CXCR4-tropic strains (n = 33). The overall height of the stack indicates the sequence conservation at that position, while the height of the symbols within the stack indicates the relative frequency of each amino acid at that position.

Given that the emergence of X4 viruses is correlated with disease progression in subtype B infection, and that, although rare, several dual-tropic and X4-tropic viruses have been reported in subtype C from within and outside India, we sought to understand the relative magnitude of genetic variation between the V3 amino acid sequences of the predicted X4- and R5-tropic strains of the Indian origin. Consensus sequence logos for V3 amino acid sequences of 1012 R5-and 33 X4-tropic strains were determined using WebLogo v.3 http://weblogo.berkeley.edu/logo.cgi. This analysis identified a high degree of conservation within the key amino acid residues of V3 loop of the R5-tropic (Figure 2A), but not X4-tropic strains (Figure 2B). The V3 loop amino acid residues of the X4-tropic strains were highly variable (Figure 2B). An analysis of the V3 sequences of Indian subtype C, subtype B and subtype A/A1 irrespective of co-receptor tropism detected greater magnitude of sequence diversity in subtype B and A/A1 compared to V3 sequences of Indian HIV-1 subtype C (see additional file 3).

## Discussion

The overwhelming majority of Indian HIV-1 subtype C strains, 96.8% (1012 out of 1045) were predicted to be R5-tropic according to C-PSSM analysis. There were discrepancies in predicting HIV-1 subtype C X4-tropism as most of the tools were developed for HIV-1 subtype B strains. The C-PSSM represents an improvement over currently available methods for predicting X4-viruses in subtype C populations; this method has an estimated sensitivity of 81.8% and specificity of 93.3%, values considerably superior compared to other tools [8]. Investigators studying co-receptor tropism prediction among African subtype C strains reported 100% concordance of C-PSSM with phenotypic assay in detecting CXCR4 [12]. Our data supports the predominance of R5 phenotype in subtype C infected patients in India. Our data also revealed a correlation between X4-tropism and the duration since first diagnosis although this is to be interpreted with caution given that only 2 X4-tropic strains were studied. Both patients harboring probable X4-tropic virus were diagnosed with HIV-1 infection for longer than 5 years. Two previous reports of X4-tropic HIV-1 subtype C viruses from India have not commented on the length of infection [19,20]. The presence of X4-tropic strains is well known to be significantly associated with longer duration of HIV infection [27]. Variations at position 16 and 18 of the V3 loop in R5 viruses have been reported to lead to X4 tropism [28]. In all the Indian R5 tropic HIV-1 subtype C sequences including cohort sequences (additional file 2), the GPGQ crown motif was significantly conserved, but in Indian X4 tropic strains, the 18th position was variable (Figure 2B). The overall conserved nature of HIV-1 subtype C V3 sequence may reduce the possibility of co-receptor switch in subtype C viruses and may partially explain the low prevalence of X4-tropic strains. An additional contributory factor for the high prevalence of R5-tropic strains may be the presence of a large pool of CCR5 positive CD4 cells in the Indian population which allows for the R5-strains to have improved replication fitness [29]. CCR5 expression on CD4+ cells of HIV-1 infected individuals is higher among the Indian population (26.8%) [29] compared to the population in the USA (13.2%) [30]. Two European studies showed varying levels of expression of CCR5 on CD4+ cells from HIV-infected individuals; 28% in Netherlands [31] and 17% in Italy [32].

The implementation of antiretroviral therapy (ART) in resource-limited settings requires use of standard first- and second-line therapies. CCR5 receptor antagonists such as maraviroc is a potential future option for second-line therapy in populations where R5-tropic strains predominate [33,34]. The high proportion of R5-tropic strains and decreased evidence of co-receptor switch in HIV-1 subtype C viruses in India support the proposition that CCR5-antagonists may be promising drugs for future HIV treatment although concerns about potential overgrowth of X4-tropic strains need to be adequately addressed.

## Conclusions

The present study, the first of its kind from India where a large number of *env *sequences were subjected to *in silico *co-receptor prediction analysis, revealed high prevalence of R5-tropism and greater homogeneity within the V3 loop sequences of HIV-1 subtype C Indian strains. Although prediction tools may not entirely substitute experimental evaluation, the simplicity of *in silico *strategies highlighted in this study can be a major advantage for coreceptor tropism prediction in resource-constrained settings. Furthermore, our findings also allude to the possibility of including CCR5 antagonists to the anti-retroviral repertoire with additional necessary precautions. The therapeutic implications of our findings are of global relevance and will facilitate further research on HIV-1 co-receptor usage and viral diversity.

## Conflicts of interests

The authors declare that they have no competing interests.

## Authors' contributions

UN designed the study, performed all laboratory tests and bioinformatics analysis. UN, ADC and AS drafted the manuscript. UN, AS, GDS, PBS obtained ethical approval and helped with subject recruitment. AS, VSK, GDS and PBS provided clinical expertise and UR provided scientific guidance. All authors have read and approved the final manuscript.

## Supplementary Material

Additional file 1 **Indian V3 sequences used in this study**. Accession numbers and sequence information of Indian V3 region was given in multiple alignment format. Sequences were downloaded from Los Alamos Database accessed on 7 Feb 2010. Click here for file

Additional file 2 **Multiple sequence analysis of Env V3 region of clinical isolates**: Multiple sequence analysis was carried out in ClustalW. Dots represent residual similarity with consensus C sequences downloaded from Los Alamos Database. Dash indicates deletion in that position. Click here for file

Additional file 3 **Subtype specific Consensus sequence logo**. Consensus sequence logos of **A**. Subtype C strains (n = 1045), **B**. Subtype B (n = 56) and **C**. Subtype A/A1 strains (n = 17) irrespective of the co-receptor tropism. Consensus sequence logos were created using WebLogo ver 3 http://weblogo.berkeley.edu/logo.cgi. Click here for file
