# Supplementary material for: Co-receptor tropism prediction among 1045 Indian HIV-1 subtype C sequences: Therapeutic implications for India
Source: AIDS Res Ther. 2010 Jul 21;7:24. doi: 10.1186/1742-6405-7-24 (PMC2918521; doi:10.1186/1742-6405-7-24)
Supplement: Additional file 1 — Indian V3 sequences used in this study. Accession numbers and sequence information of Indian V3 region was given in multiple alignment format. Sequences were downloaded from Los Alamos Database accessed on 7 Feb 2010. [file 1742-6405-7-24-S1.DOC]

....|....| ....|....| ....|....| ....|

5 15 25 35

AF067159 CTRPNNNTRK SIRIGPGQTF YATGEIVGNI RQAHC

AY669746 CTRPNNNTRK SIRIGPGQTF YATGEIVGNI RQAHC

AF392581 CTRPNNNTRK SIRIGPGQTF YATGEIIGNI RQAHC

AF392582 CTRPNNNTRK SIRIGPGQTF YATGEIIGNI RQAHC

DQ325301 CTRPNNNTRK SIRIGPGQTF YATGEIIGNI RQAHC

EF117265 CTRPNNNTRK SIRIGPGQTF YATGEIIGNI RQAHC

EU781867 CTRPNNNTRK SIRIGPGQTF YATGEIIGNI RQAHC

AY525929 CTRPNNNTRK SIRIGPGQTF YAPGEVVGDI RQAHC

AY567526 RTRPNNNTRK SIRIGPGQTF YATGEVIGDI GQAHC

AY567516 CTRPHNNTRK SIKIGPGQTF YATGEVIGDI RQAHC

DQ325305 CTRPHNNKRK SIRIGPGQTF YATGEVIGDI RQAHC

AY525897 CTRPNNNTRK SIRIGPGQTF YATGEVIGNI RQAHC

AF148236 CTRPNNNTRK SIRIGPGQTF YAAGEIIGNI RQAHC

AF148237 CTRPNNNTRK SIRIGPGQTF YAAGDIIGNI RQAHC

AF148238 CTRPNNNTRK SIRIGPGQTF YATGYIIGNI RQAHC

EU781887 CTRPNNNTRK SIRIGPGQTF YATGQIIGNI RQAHC

AF250240 CTRPNNNTRK SIRIGPGQTF YATGAIIGNI RQAHC

AY208967 CTRPNNNTRK SIRIGPGQTF YATGAIIGNI RQAHC

AJ311641 CTRPNNNTRK SIRIGPGQTF YATGDIIGNI RQAYC

AY128265 CTRPNNNTRK SIRIGPGQTF YATGDIIGNI RQAYC

EF117268 CTRPNNNTRK SIRIGPGQTF YATGDIIGNI RQAYC

EU908224 CTRPNNNTRK SIRIGPGQTF YATGDIIGNI RQAYC

FJ541990 CTRPNNNTRK SIRIGPGQTF YATGDIIGNI RQAYC

DQ325329 CTRPNNNTRK SIRIGPGRTF YATGDIIGNI RQAYC

AF250256 CTRPNNNTRK SIRIGPGQTF YATGDITGNI RQAYC

DQ381971 CTRPNNNTRR SIRIGPGQTF YATGDIIGNI RHAYC

EU908225 CTRPNNNTRR SIRIGPGQTF YATGDIIGNI RQAYC

AY128264 CTRPNNNTRG SIRIGPGQTF YATGDIIGNI RQAYC

AJ311643 CTRPHNNTRK SIRIGPGQTF YATGDIIGNI RQAYC

AF392583 CTRPNNNTRK SIRIGPGQTF YATGEIIGNI RQAYC

AF392584 CTRPNNNTRK SIRIGPGQTF YATGEIIGNI RQAYC

FJ541933 CTRPNNNTRK SIRIGPGQTF YATGEIIGNI RQAYC

FJ541934 CTRPNNNTRK SIRIGPGQTF YATGEIIGNI RQAYC

FJ541935 CTRPNNNTRK SIRIGPGQTF YATGEIIGNI RQAYC

FJ541936 CTRPNNNTRK SIRIGPGQTF YATGEIIGNI RQAYC

FJ541937 CTRPNNNTRK SIRIGPGQTF YATGEIIGNI RQAYC

FJ541939 CTRPNNNTRK SIRIGPGQTF YATGEIIGNI RQAYC

FJ541940 CTRPNNNTRK SIRIGPGQTF YATGEIIGNI RQAYC

FJ541941 CTRPNNNTRK SIRIGPGQTF YATGEIIGNI RQAYC

FJ541942 CTRPNNNTRK SIRIGPGQTF YATGEIIGNI RQAYC

FJ541943 CTRPNNNTRK SIRIGPGQTF YATGEIIGNI RQAYC

FJ541945 CTRPNNNTRK SIRIGPGQTF YATGEIIGNI RQAYC

FJ541946 CTRPNNNTRK SIRIGPGQTF YATGEIIGNI RQAYC

FJ541947 CTRPNNNTRK SIRIGPGQTF YATGEIIGNI RQAYC

FJ541950 CTRPNNNTRK SIRIGPGQTF YATGEIIGNI RQAYC

FJ541952 CTRPNNNTRK SIRIGPGQTF YATGEIIGNI RQAYC

FJ541954 CTRPNNNTRK SIRIGPGQTF YATGEIIGNI RQAYC

FJ541955 CTRPNNNTRK SIRIGPGQTF YATGEIIGNI RQAYC

FJ541956 CTRPNNNTRK SIRIGPGQTF YATGEIIGNI RQAYC

FJ541963 CTRPNNNTRK SIRIGPGQTF YATGEIIGNI RQAYC

FJ541964 CTRPNNNTRK SIRIGPGQTF YATGEIIGNI RQAYC

FJ541965 CTRPNNNTRK SIRIGPGQTF YATGEIIGNI RQAYC

FJ541972 CTRPNNNTRK SIRIGPGQTF YATGEIIGNI RQAYC

FJ541973 CTRPNNNTRK SIRIGPGQTF YATGEIIGNI RQAYC

FJ541974 CTRPNNNTRK SIRIGPGQTF YATGEIIGNI RQAYC

FJ541975 CTRPNNNTRK SIRIGPGQTF YATGEIIGNI RQAYC

FJ541977 CTRPNNNTRK SIRIGPGQTF YATGEIIGNI RQAYC

FJ541986 CTRPNNNTRK SIRIGPGQTF YATGEIIGNI RQAYC

FJ541988 CTRPNNNTRK SIRIGPGQTF YATGEIIGNI RQAYC

FJ541938 CTRPNNNTRK SIGIGPGQTF YATGEIIGNI RQAYC

FJ541971 CTRPNNSTRK SIRIGPGQTF YATGEIIGNI RQAYC

FJ541944 CTRPNNNTRK SIRVGPGQTF YATGEIIGNI RQAYC

FJ541951 CTRPNNNIRK SIRIGPGQTF YATGEIIGNI RQAYC

DQ325326 CTRPNNNTRK SIRIGPGQTF YATGSIIGNI RQAYC

U53283 CTRPNNNTRK SIRIGPGQTF YATGNIIGNI RQAYC

AJ278384 CTRPNNNTRK SIRIGPGQTF YATGAIIGNI RQAYC

AJ292008 CTRPNNNTRK SIRIGPGQTF YATGAIIGNI RQA--

AF148239 CTRPNNNTRK SIRIGPGQTF YATGDIIGNI RQAHC

AF148243 CTRPNNNTRK SIRIGPGQTF YATGDIIGNI RQAHC

AF392585 CTRPNNNTRK SIRIGPGQTF YATGDIIGNI RQAHC

AY653068 CTRPNNNTRK SIRIGPGQTF YATGDIIGNI RQAHC

DQ367251 CTRPNNNTRK SIRIGPGQTF YATGDIIGNI RQAHC

EF469243 CTRPNNNTRK SIRIGPGQTF YATGDIIGNI RQAHC

U53281 CTRPNNNTRK SIRIGPGQTF YATGDIIGNI RQAHC

U53296 CTRPNNNTRK SIRIGPGQTF YATGDIIGNI RQAHC

U53298 CTRPNNNTRK SIRIGPGQTF YATGDIIGNI RQAHC

U53299 CTRPNNNTRK SIRIGPGQTF YATGDIIGNI RQAHC

AF148241 CTRPSNNTRK SIRIGPGQTF YATGDIIGNI REAYC

AY525873 CTRPSNNTRK SIRIGPGQIF YATGDIIGNI REAHC

AJ278383 CTRPSNNTRK SIRIGPGQTF YATGDIIGNI RGAHC

AY064244 CTRPSNNTRK SIRIGPGQTF YATGDIIGNI RQAHC

DQ149146 CTRPSNNTRK SIRIGPGQTF YATGEIIGNI RQAHC

AY525850 CTRPGNNTRK SIRIGPGQTF YATGDIIGNI RQAHC

AY525872 CTRPGNNTRK SIRIGPGQTF YATGDIIGNI RQAHC

AY525878 CTRPGNNTRK SIRIGPGQTF YATGDIIGNI RQAHC

EU908222 CTRPGNNTRK SIRIGPGQTF YATGDIIGNI RQAHC

EU908223 CTRPGNNTRK SIRIGPGQTF YATGDIIGNI RQAHC

EF117274 CTRPGNNTRK SIRIGPGQTF YATGEIIGNI RQAHC

FJ541996 CTRPGNNTRK SVRIGPGQTF YATGEIIGNI RQAHC

EU908216 CTRPGNNTRK SIRIGPGQTF YATGGIIGNI RQAHC

EU908217 CTRPGNNTRK SIRIGPGQTF YATGGIIGNI RQAHC

FJ968671 CIRPSNNTRK SIRIGPGQAF YATGGIIGNI RQAHC

FJ968672 CIRPSNNTRK SIRIGPGQAF YATGGIIGNI RQAHC

FJ769835 CIRPSNNTRK SIRTGPGQAF YATGGIIGNI RQAHC

GU057985 CIRPNNNTRK SIRIGPGQAF YATGDIIGNI RQAHC

GU071077 CIRPNNNTRK SIRIGPGQAF YATGDIIGNI RQAHC

GU071079 CIRPNNNTRK SIRIGPGQAF YATGDIIGNI RQAHC

GU071080 CIRPNNNTRK SIRIGPGQAF YATGDIIGNI RQAHC

GU071082 CIRPNNNTRK SIRIGPGQAF YATGDIIGNI RQAHC

GU117712 CIRPNNNTRK SIRIGPGQAF YATGDIIGNI RQAHC

AF392567 CIRPNNNTRK SIRIGPGQTF YATGDIIGNI RQAHC

DQ325303 CVRPNNNTRK SIRIGPGQTF YATGDIIGNI RQAHC

EU781892 CVRPGNNTRK SIRIGPGQTF YATGDIIGNI RQAHC

AF067154 CVRPNNNTRE SIRIGPGQTF YATGEIIGDI RQAHC

AY669743 CVRPNNNTRE SIRIGPGQTF YATGEIIGDI RQAHC

AJ292010 CVRPNNNTRK SIRIGPGQTF YATGEIIGDI RQAHC

EU781855 CARPNNNTRK SIRIGPGQTF YATGEIIGDI RQAHC

U53294 CARPNNNTRK SIRIGPGQTF YATGGIIGDI RQAHC

AF067157 CTRPNNNTRK SIRIGPGQTF YATGEIIGDI RQAHC

AF148256 CTRPNNNTRK SIRIGPGQTF YATGEIIGDI RQAHC

AF250248 CTRPNNNTRK SIRIGPGQTF YATGEIIGDI RQAHC

AF250253 CTRPNNNTRK SIRIGPGQTF YATGEIIGDI RQAHC

AF250254 CTRPNNNTRK SIRIGPGQTF YATGEIIGDI RQAHC

AF392586 CTRPNNNTRK SIRIGPGQTF YATGEIIGDI RQAHC

AF392604 CTRPNNNTRK SIRIGPGQTF YATGEIIGDI RQAHC

AJ278375 CTRPNNNTRK SIRIGPGQTF YATGEIIGDI RQAHC

AY525864 CTRPNNNTRK SIRIGPGQTF YATGEIIGDI RQAHC

DQ149144 CTRPNNNTRK SIRIGPGQTF YATGEIIGDI RQAHC

EU526664 CTRPNNNTRK SIRIGPGQTF YATGEIIGDI RQAHC

EU781872 CTRPNNNTRK SIRIGPGQTF YATGEIIGDI RQAHC

FJ541948 CTRPNNNTRK SIRIGPGQTF YATGEIIGDI RQAHC

U53278 CTRPNNNTRK SIRIGPGQTF YATGEIIGDI RQAHC

U53288 CTRPNNNTRK SIRIGPGQTF YATGEIIGDI RQAHC

U53295 CTRPNNNTRK SIRIGPGQTF YATGEIIGDI RQAHC

AF148260 CTRPSNNTRK SIRIGPGQTF YATGEIIGDI RQAHC

AF250257 CTRPSNNTRK SIRIGPGQTF YATGEIIGDI RQAHC

AY567512 CTRPSNNTRK SIRIGPGQTF YATGEIIGDI RQAHC

EU526661 CTRPSNNTRK SIRIGPGQTF YATGEIIGDI RQAHC

U07100 CTRPSNNTRK SIRIGPGQTF YATGEIIGDI RQAHC

AF148231 CTRPSNNTRK SIRIGPGQTF YATGEIIGDI RQAYC

AY567499 CTRPGNNTRK SIRIGPGQTF YATGEIIGDI RQAHC

DQ149151 CTRPGNNTRK SIRIGPGQTF YATGEIIGDI RQAHC

AF392555 CTRPHNNTRK SIRIGPGQTF YATGEIIGDV RQAHC

AF392556 CTRPHNNTRK SIRIGPGQTF YATGEIIGDV RQAHC

AF392557 CTRPHNNTRK SIRIGPGQTF YATGEIIGDV RQAHC

AF392558 CTRPHNNTRK SIRIGPGQTF YATGEIIGDV RQAHC

FJ541581 CTRPNNNTRK SIRIGPGQTF FATGEIIGDV RQAHC

AF101118 CTRPNNNTRK SIRIGPGQTF YATGGIIGNI RQAHC

AF101119 CTRPNNNTRK SIRIGPGQTF YATGGIIGNI RQAHC

AF286231 CTRPNNNTRK SIRIGPGQTF YATGGIIGNI RQAHC

AY525882 CTRPNNNTRK SIRIGPGQTF YATGGIIGRI RQAHC

AY525883 CTRPNNNTRK SIRIGPGQTF YATGGIIGRI RQAHC

AF392600 CTRPNNNTRK SIKIGPGQTF YATEGIIRDI RQAHC

AF392601 CTRPNNNTRK SIKIGPGQTF YATEGIIRDI RQAHC

EU781860 CTRPNNNTRK SIRIGPGQTF YATEGIIGNI RQAHC

AF148251 CTRPNNNTRK SIRIGPGQTF YATGGIIGDI RQAHC

AJ291688 CTRPNNNTRK SIRIGPGQTF YATGGIIGDI RQAHC

D13420 CTRPNNNTRK SIRIGPGQTF YATGGIIGDI RQAHC

U53303 CTRPNNNTRK SIRIGPGQTF YATGGIIGDI RQAHC

AY653044 CTRPNNNTRK SIRIGPGQTF YATGGGIGDI RQAHC

AF148244 CTRPNNNTRK SIRIGPGQTF YATGAIIGDI RQAHC

AY567521 CTRPNNNTRK SIRIGPGQTF YATGAIIGDI RQAHC

DQ367249 CTRPNNNTRK SIRIGPGQTF YATGAIIGDI RQAHC

AY525860 CTRPNNNTRK SIRIGPGQTF YATGAIIGDI REAHC

AY567529 CTRPNNNTRK SIRIGPGQTF YATGAIIGDI KEAHC

AJ278380 CTRPNNNTRK SIRIGPGQTF YATGAIIGDI RPAHC

AF250260 CTRPNNNTRK SIRIGPGQTF FATGDIIGDI RQAHC

AY208968 CTRPNNNTRK SIRIGPGQTF FATGDIIGDI RQAHC

DQ325309 CTRPNNNTRK SIRIGPGQTF FATGDIIGDI RQAHC

DQ325310 CTRPNNNTRK SIRIGPGQTF FATGDIIGDI RQAHC

DQ325311 CTRPNNNTRK SIRIGPGQTF FATGDIIGDI RQAHC

DQ325316 CTRPNNNTRK SIRIGPGQTF FATGDIIGDI RQAHC

DQ325317 CTRPNNNTRK SIRIGPGQTF FATGDIIGDI RQAHC

EU781870 CTRPNNNTRK SIRIGPGQTF FATGDIIGDI RQAHC

DQ325320 CTRPNNNTRK SMRIGPGQTF FATGDIIGDI RQAHC

AF250243 CTRPNNNTRK SIRIGPGQTF FATGDIIGRI RQAHC

DQ149142 CTRPNNNTRE SIRIGPGQTF FATGDIIGDI RQAHC

FJ541613 CTRPNNNTRE SIRIGPGQTF FATGEIIGDI RQAHC

DQ325321 CARPNNNTRK SVRIGPGQTF FATGEIIGDI RQAHC

DQ367256 CTRPNNNTRR SVRIGPGQTF FATGEIIGDI RQAYC

FJ541631 CTRPNNNTRK STRIGPGQTF FATGEIIGDI RQAHC

FJ541632 CTRPNNNTRK STRIGPGQTF FATGEIIGDI RQAHC

FJ541635 CTRPNNNTRK GTRIGPGQTF FATGEIIGDI RQAHC

FJ541526 CTRPNNNTRK STRIGPGQTF FAQGEIIGDI RQAHC

FJ541602 CTRPNNNTRK SRRIGPGQTF FATGEIIGDI RQAHC

FJ541567 CTRPNNNTRK SIRIGPGQTF FATGEIIGDI RQAHC

FJ541568 CTRPNNNTRK SIRIGPGQTF FATGEIIGDI RQAHC

FJ541570 CTRPNNNTRK SIRIGPGQTF FATGEIIGDI RQAHC

FJ541571 CTRPNNNTRK SIRIGPGQTF FATGEIIGDI RQAHC

FJ541572 CTRPNNNTRK SIRIGPGQTF FATGEIIGDI RQAHC

FJ541573 CTRPNNNTRK SIRIGPGQTF FATGEIIGDI RQAHC

FJ541575 CTRPNNNTRK SIRIGPGQTF FATGEIIGDI RQAHC

FJ541576 CTRPNNNTRK SIRIGPGQTF FATGEIIGDI RQAHC

FJ541577 CTRPNNNTRK SIRIGPGQTF FATGEIIGDI RQAHC

FJ541578 CTRPNNNTRK SIRIGPGQTF FATGEIIGDI RQAHC

FJ541579 CTRPNNNTRK SIRIGPGQTF FATGEIIGDI RQAHC

FJ541580 CTRPNNNTRK SIRIGPGQTF FATGEIIGDI RQAHC

FJ541582 CTRPNNNTRK SIRIGPGQTF FATGEIIGDI RQAHC

FJ541583 CTRPNNNTRK SIRIGPGQTF FATGEIIGDI RQAHC

FJ541584 CTRPNNNTRK SIRIGPGQTF FATGEIIGDI RQAHC

FJ541585 CTRPNNNTRK SIRIGPGQTF FATGEIIGDI RQAHC

FJ541586 CTRPNNNTRK SIRIGPGQTF FATGEIIGDI RQAHC

FJ541587 CTRPNNNTRK SIRIGPGQTF FATGEIIGDI RQAHC

FJ541589 CTRPNNNTRK SIRIGPGQTF FATGEIIGDI RQAHC

FJ541590 CTRPNNNTRK SIRIGPGQTF FATGEIIGDI RQAHC

FJ541591 CTRPNNNTRK SIRIGPGQTF FATGEIIGDI RQAHC

FJ541592 CTRPNNNTRK SIRIGPGQTF FATGEIIGDI RQAHC

FJ541593 CTRPNNNTRK SIRIGPGQTF FATGEIIGDI RQAHC

FJ541594 CTRPNNNTRK SIRIGPGQTF FATGEIIGDI RQAHC

FJ541595 CTRPNNNTRK SIRIGPGQTF FATGEIIGDI RQAHC

FJ541596 CTRPNNNTRK SIRIGPGQTF FATGEIIGDI RQAHC

FJ541597 CTRPNNNTRK SIRIGPGQTF FATGEIIGDI RQAHC

FJ541598 CTRPNNNTRK SIRIGPGQTF FATGEIIGDI RQAHC

FJ541599 CTRPNNNTRK SIRIGPGQTF FATGEIIGDI RQAHC

FJ541600 CTRPNNNTRK SIRIGPGQTF FATGEIIGDI RQAHC

FJ541601 CTRPNNNTRK SIRIGPGQTF FATGEIIGDI RQAHC

FJ541603 CTRPNNNTRK SIRIGPGQTF FATGEIIGDI RQAHC

FJ541605 CTRPNNNTRK SIRIGPGQTF FATGEIIGDI RQAHC

FJ541606 CTRPNNNTRK SIRIGPGQTF FATGEIIGDI RQAHC

FJ541607 CTRPNNNTRK SIRIGPGQTF FATGEIIGDI RQAHC

FJ541608 CTRPNNNTRK SIRIGPGQTF FATGEIIGDI RQAHC

FJ541609 CTRPNNNTRK SIRIGPGQTF FATGEIIGDI RQAHC

FJ541611 CTRPNNNTRK SIRIGPGQTF FATGEIIGDI RQAHC

FJ541612 CTRPNNNTRK SIRIGPGQTF FATGEIIGDI RQAHC

FJ541614 CTRPNNNTRK SIRIGPGQTF FATGEIIGDI RQAHC

FJ541615 CTRPNNNTRK SIRIGPGQTF FATGEIIGDI RQAHC

FJ541617 CTRPNNNTRK SIRIGPGQTF FATGEIIGDI RQAHC

FJ541618 CTRPNNNTRK SIRIGPGQTF FATGEIIGDI RQAHC

FJ541620 CTRPNNNTRK SIRIGPGQTF FATGEIIGDI RQAHC

FJ541622 CTRPNNNTRK SIRIGPGQTF FATGEIIGDI RQAHC

FJ541624 CTRPNNNTRK SIRIGPGQTF FATGEIIGDI RQAHC

FJ541625 CTRPNNNTRK SIRIGPGQTF FATGEIIGDI RQAHC

FJ541626 CTRPNNNTRK SIRIGPGQTF FATGEIIGDI RQAHC

FJ541627 CTRPNNNTRK SIRIGPGQTF FATGEIIGDI RQAHC

FJ541628 CTRPNNNTRK SIRIGPGQTF FATGEIIGDI RQAHC

FJ541629 CTRPNNNTRK SIRIGPGQTF FATGEIIGDI RQAHC

FJ541630 CTRPNNNTRK SIRIGPGQTF FATGEIIGDI RQAHC

FJ541633 CTRPNNNTRK SIRIGPGQTF FATGEIIGDI RQAHC

FJ541636 CTRPNNNTRK SIRIGPGQTF FATGEIIGDI RQAHC

FJ541637 CTRPNNNTRK SIRIGPGQTF FATGEIIGDI RQAHC

FJ541638 CTRPNNNTRK SIRIGPGQTF FATGEIIGDI RQAHC

FJ541639 CTRPNNNTRK SIRIGPGQTF FATGEIIGDI RQAHC

FJ541640 CTRPNNNTRK SIRIGPGQTF FATGEIIGDI RQAHC

FJ541634 CTRSNNNTRK SIRIGPGQTF FATGEIIGDI RQAHC

FJ541574 CTRPNYNTRK SIRIGPGQTF FATGEIIGDI RQAHC

FJ541616 CTRPNDNTRK SIRIGPGQTF FATGEIIGDI RQAHC

FJ541604 CTRPNNNTRK SIRIGPGQTF FATGEIIRDI RQAHC

FJ541621 CTRPNNNTRK SIRIGPGHTF FATGEIIGDI RQAHC

FJ541569 CTRPNNNTRK SIRIGPGQTL FATGEIIGDI RQAHC

FJ541588 CTRPNNNTRK SIRIGPGQTL FATGEIIGDI RQAHC

FJ541623 CTRPNNNTRK SIRIGPGQTF FATGEMIGDI RQAHC

FJ541619 CTRPNNNTRK SIRIGPGQTF FATGEITGDI RQAHC

FJ541508 CTRPNNNTRK SIRIGPGQTF FAQGEIIGDI RQAHC

FJ541509 CTRPNNNTRK SIRIGPGQTF FAQGEIIGDI RQAHC

FJ541510 CTRPNNNTRK SIRIGPGQTF FAQGEIIGDI RQAHC

FJ541511 CTRPNNNTRK SIRIGPGQTF FAQGEIIGDI RQAHC

FJ541513 CTRPNNNTRK SIRIGPGQTF FAQGEIIGDI RQAHC

FJ541514 CTRPNNNTRK SIRIGPGQTF FAQGEIIGDI RQAHC

FJ541515 CTRPNNNTRK SIRIGPGQTF FAQGEIIGDI RQAHC

FJ541516 CTRPNNNTRK SIRIGPGQTF FAQGEIIGDI RQAHC

FJ541517 CTRPNNNTRK SIRIGPGQTF FAQGEIIGDI RQAHC

FJ541518 CTRPNNNTRK SIRIGPGQTF FAQGEIIGDI RQAHC

FJ541519 CTRPNNNTRK SIRIGPGQTF FAQGEIIGDI RQAHC

FJ541520 CTRPNNNTRK SIRIGPGQTF FAQGEIIGDI RQAHC

FJ541521 CTRPNNNTRK SIRIGPGQTF FAQGEIIGDI RQAHC

FJ541522 CTRPNNNTRK SIRIGPGQTF FAQGEIIGDI RQAHC

FJ541523 CTRPNNNTRK SIRIGPGQTF FAQGEIIGDI RQAHC

FJ541524 CTRPNNNTRK SIRIGPGQTF FAQGEIIGDI RQAHC

FJ541527 CTRPNNNTRK SIRIGPGQTF FAQGEIIGDI RQAHC

FJ541528 CTRPNNNTRK SIRIGPGQTF FAQGEIIGDI RQAHC

FJ541529 CTRPNNNTRK SIRIGPGQTF FAQGEIIGDI RQAHC

FJ541530 CTRPNNNTRK SIRIGPGQTF FAQGEIIGDI RQAHC

FJ541531 CTRPNNNTRK SIRIGPGQTF FAQGEIIGDI RQAHC

FJ541532 CTRPNNNTRK SIRIGPGQTF FAQGEIIGDI RQAHC

FJ541533 CTRPNNNTRK SIRIGPGQTF FAQGEIIGDI RQAHC

FJ541534 CTRPNNNTRK SIRIGPGQTF FAQGEIIGDI RQAHC

FJ541535 CTRPNNNTRK SIRIGPGQTF FAQGEIIGDI RQAHC

FJ541536 CTRPNNNTRK SIRIGPGQTF FAQGEIIGDI RQAHC

FJ541537 CTRPNNNTRK SIRIGPGQTF FAQGEIIGDI RQAHC

FJ541538 CTRPNNNTRK SIRIGPGQTF FAQGEIIGDI RQAHC

FJ541539 CTRPNNNTRK SIRIGPGQTF FAQGEIIGDI RQAHC

FJ541540 CTRPNNNTRK SIRIGPGQTF FAQGEIIGDI RQAHC

FJ541541 CTRPNNNTRK SIRIGPGQTF FAQGEIIGDI RQAHC

FJ541542 CTRPNNNTRK SIRIGPGQTF FAQGEIIGDI RQAHC

FJ541544 CTRPNNNTRK SIRIGPGQTF FAQGEIIGDI RQAHC

FJ541545 CTRPNNNTRK SIRIGPGQTF FAQGEIIGDI RQAHC

FJ541546 CTRPNNNTRK SIRIGPGQTF FAQGEIIGDI RQAHC

FJ541547 CTRPNNNTRK SIRIGPGQTF FAQGEIIGDI RQAHC

FJ541548 CTRPNNNTRK SIRIGPGQTF FAQGEIIGDI RQAHC

FJ541550 CTRPNNNTRK SIRIGPGQTF FAQGEIIGDI RQAHC

FJ541551 CTRPNNNTRK SIRIGPGQTF FAQGEIIGDI RQAHC

FJ541552 CTRPNNNTRK SIRIGPGQTF FAQGEIIGDI RQAHC

FJ541553 CTRPNNNTRK SIRIGPGQTF FAQGEIIGDI RQAHC

FJ541554 CTRPNNNTRK SIRIGPGQTF FAQGEIIGDI RQAHC

FJ541555 CTRPNNNTRK SIRIGPGQTF FAQGEIIGDI RQAHC

FJ541556 CTRPNNNTRK SIRIGPGQTF FAQGEIIGDI RQAHC

FJ541557 CTRPNNNTRK SIRIGPGQTF FAQGEIIGDI RQAHC

FJ541558 CTRPNNNTRK SIRIGPGQTF FAQGEIIGDI RQAHC

FJ541559 CTRPNNNTRK SIRIGPGQTF FAQGEIIGDI RQAHC

FJ541560 CTRPNNNTRK SIRIGPGQTF FAQGEIIGDI RQAHC

FJ541561 CTRPNNNTRK SIRIGPGQTF FAQGEIIGDI RQAHC

FJ541562 CTRPNNNTRK SIRIGPGQTF FAQGEIIGDI RQAHC

FJ541563 CTRPNNNTRK SIRIGPGQTF FAQGEIIGDI RQAHC

FJ541564 CTRPNNNTRK SIRIGPGQTF FAQGEIIGDI RQAHC

FJ541565 CTRPNNNTRK SIRIGPGQTF FAQGEIIGDI RQAHC

FJ541566 CTRPNNNTRK SIGIGPGQTF FAQGEIIGDI RQAHR

FJ541543 CTRPNNNTRK SIRIGPGQTF FAQGEIKGDI RQAHC

FJ541512 CTRPNNNTRR SIRIGPGQTF FAQGEIIGDI RQAHC

FJ541525 CTRPSNNTRK SIRIGPGQTF FAQGEIIGDI RQAHC

FJ541549 CTRPNNNTRK SIRIGPGQTF SAQGEIIGDI RQAHC

FJ541610 CTRPNNNTRK SIRIGPGQTF FATGKIIGDI RQAHC

A37291 YTRPNNNTRK GVRIGPGQTF YATGDIIGDI RQAHC

L07653 YTRPNNNTRK GVRIGPGQTF YATGDIIGDI RQAHC

X65638 YTRPNNNTRK GVRIGPGQTF YATGDIIGDI RQAHC

AF250246 CTRPNNNTRK GIRIGPGQTF YATGDIIGDI RQAHC

AF101125 CTRPNNNTRK SVRIGPGQTF YATGDIIGDI RQAYC

AF392589 CTRPNNNTRK SVRIGPGQTF YATGDIIGDI RQAYC

AY525899 CTRPNNNTRK SVRIGPGQTF YATGDIIGDI RQAYC

FJ541979 CTRPNNNIRK GVRIGPGQTF YATGDIIGDI RQAYC

FJ541995 CTRPNNNIRK SVRIGPGQTF YATGDIIGDI RQAYC

FJ541978 CTRPNNNIRK SVRIGPGQTF YATGDTIGDI RQAYC

DQ381979 CIRPNNNIRK SVRIGPGQTF YATGDIIGDI RQAHC

AF148234 CTRPNNNTRK SVRIGPGQTF YATGEIIGDI RQAYC

EU526660 CTRPNNNTRK GVRVGPGQMF YATGEIIGDI RQAYC

AF392577 CTRPNNNTRK SVRIGPGQVF YATGEIIGDI RQAHC

EF117270 CTRPNNNTRK SVRIGPGQTF YATGEIIGDI RQAHC

AF148261 CTRPNNNTRK SVRIGPGQTF YATGDIIGDI RQAHC

AY653047 CTRPNNNTRK SVRIGPGQTF YATGDIIGDI RQAHC

AY653071 CTRPNNNTRK SVRIGPGQTF YATGDIIGDI RQAHC

DQ381977 CTRPNNNTRK SVRIGPGQTF YATGDIIGDI RQAHC

AF250259 CTRPGNNTRK SVRIGPGQTF YATGDIIGDI RQAHC

FJ541949 CTRPGNNTRK SVRIGPGQTF YATGDIIGDI RQAHC

EU781866 CTRPGNNTRK SVRIGPGQTF YATGDIIGDI RKAHC

EU781877 CTRPGNNTRK SVRIGPGQTF YATGDIIGDI RRAHC

AY567509 CSRPSNNTRK SVRIGPGQTF YATGDIIGDI RRAHC

DQ149143 CTRASNNTRK SVRIGPGQTF YATGDIIGDI RQAHC

EU781890 CTRPSNNTRK SVRIGPGQTF YATGDIIGDI RQAHC

DQ149149 CTRPSNNTRK SVRIGPGQTF YATGDIIGNI RQAHC

AF392576 CTRPTNNTRK SVRIGPGQMF YATGDIIGDI RQAHC

AY567508 CTRPNNNTRK SVRIGPGQIF YATGDIIGDI RQAHC

FJ541716 CTRPNNNTRT SVRIGPGQTF YATGDIIGDP RQAHC

FJ541725 CTRPNNNTRT SVRIGPGQTF YATGDIIGDP RQAHC

FJ541736 CTRPNNNTRT SVRIGPGQTF YATGDIIGDP RQAHC

FJ541748 CTRPNNNTRT SVRIGPGQTF YATGDIIGDP RQAHC

FJ541751 CTRPNNNTRT SVRIGPGQTF YATGDIIGDP RQAHC

FJ541752 CTRPNNNTRT SVRIGPGQTF YATGDIIGDP RQAHC

FJ541753 CTRPNNNTRT SVRIGPGQTF YATGDIIGDP RQAHC

FJ541754 CTRPNNNTRT SVRIGPGQTF YATGDIIGDP RQAHC

FJ541755 CTRPNNNTRT SVRIGPGQTF YATGDIIGDP RQAHC

FJ541783 CTRPNNNTRT SVRIGPGQTF YATGDIIGDP RQAHC

FJ541784 CTRPNNNTRT SVRIGPGQTF YATGDIIGDP RQAHC

FJ541785 CTRPNNNTRT SVRIGPGQTF YATGDIIGDP RQAHC

FJ541787 CTRPNNNTRT SVRIGPGQTF YATGDIIGDP RQAHC

FJ541780 CTRPNNNTRT SVRIRPGQTF YATGDIIGDP RQAHC

FJ541721 CTRPSNNTRT SVRIGPGQTF YATGDIIGNP RQAHC

FJ541724 CTRPSNNTRT SVRIGPGQTF YATGDIIGNP RQAHC

FJ541728 CTRPSNNTRT SVRIGPGQTF YATGDIIGNP RQAHC

FJ541731 CTRPSNNTRT SVRIGPGQTF YATGDIIGNP RQAHC

FJ541735 CTRPSNNTRT SVRIGPGQTF YATGDIIGNP RQAHC

FJ541741 CTRPSNNTRT SVRIGPGQTF YATGDIIGNP RQAHC

FJ541743 CTRPSNNTRT SVRIGPGQTF YATGDIIGNP RQAHC

FJ541747 CTRPSNNTRT SVRIGPGQTF YATGDIIGNP RQAHC

FJ541760 CTRPSNNTRT SVRIGPGQTF YATGDIIGNP RQAHC

FJ541761 CTRPSNNTRT SVRIGPGQTF YATGDIIGNP RQAHC

FJ541763 CTRPSNNTRT SVRIGPGQTF YATGDIIGNP RQAHC

FJ541765 CTRPSNNTRT SVRIGPGQTF YATGDIIGNP RQAHC

FJ541766 CTRPSNNTRT SVRIGPGQTF YATGDIIGNP RQAHC

FJ541767 CTRPSNNTRT SVRIGPGQTF YATGDIIGNP RQAHC

FJ541776 CTRPSNNTRT SVRIGPGQTF YATGDIIGNP RQAHC

FJ541778 CTRPSNNTRT SVRIGPGQTF YATGDIIGNP RQAHC

FJ541786 CTRPSNNTRT SVRIGPGQTF YATGDIIGNP RQAHC

FJ541791 CTRPSNNTRT SVRIGPGQTF YATGDIIGNP RQAHC

FJ541777 CTRPSDNTRT SVRIGPGQTF YATGDIIGNP RQAHC

FJ541779 CTRPSNSTRT SVRIGPGQTF YATGDIIGNP RQAHC

FJ541764 RTRPSNNTRT SVRIGPGQTF YATGDIIGNP RQAHC

FJ541729 CTRPSNNTRT SVRIGPGQSF YATGDIIGNP RQAHC

FJ541750 CTRPSNNTRT SVRIGPGQTF YATGDIIGDP RQAHC

FJ541756 CTRPSNNTRT SVRIGPGQTF YATGDIIGDP RQAHC

FJ541757 CTRPSNNTRT SVRIGPGQTF YATGDIIGDP RQAHC

FJ541759 CTRPSNNTRT SVRIGPGQTF YATGDIIGDP RQAHC

FJ541762 CTRPSNNTRT SVRIGPGQTF YATGDIIGDP RQAHC

FJ541774 CTRPSNNTRT SVRIGPGQTF YATGDIIGDP RQAHC

FJ541789 CTRPSNNTRT SVRIGPGQTF YATGDIIGDP RQAHC

FJ541717 CTRPGNNTRT SVRIGPGQTF YATGDIIGDP RQAHC

AY653060 CTRPGNNTRT SVRIGPGQTF YATGDIIGDI RQAHC

DQ149148 CTRPNNNTRT SVRIGPGQTF YATGDIIGDI RQAHC

AY064239 CTRPNNNTRE SVRIGPGQTF YATGDIIGDI RQAHC

FJ541796 CARPNNNTRK SVRIGPGQTF YATGDIIGDI RKAHC

FJ541819 CARPNNNTRK SVRIGPGQTF YATGDIIGDI RKAHC

FJ541833 CARPNNNTRK SVRIGPGQTF YATGDIIGDI RKAHC

FJ541834 CARPNNNTRK SVRIGPGQTF YATGDIIGDI RKAHC

FJ541835 CARPNNNTRK SVRIGPGQTF YATGDIIGDI RKAHC

FJ541836 CARPNNNTRK SVRIGPGQTF YATGDIIGDI RKAHC

FJ541807 CTRPNNNTRK SVRIGPGQTF YATGDIIGDI RKAHC

EU781874 CTRPNNNTRK SVRIGPGQTF YATGDIIGEI RAAHC

DQ149140 CTRPNNNTRK SVRIGPGQTF YATGDIIGNI RQAHC

DQ149141 CTRPNNNTRK SVRIGPGQTF YATGDIIGNI RQAHC

FJ541718 CTRPNNNTRK SVRIGPGQTF YATGDIIGDP RQAHC

FJ541719 CTRPNNNTRK SVRIGPGQTF YATGDIIGDP RQAHC

FJ541726 CTRPNNNTRK SVRIGPGQTF YATGDIIGDP RQAHC

FJ541727 CTRPNNNTRK SVRIGPGQTF YATGDIIGDP RQAHC

FJ541744 CTRPNNNTRK SVRIGPGQTF YATGDIIGDP RQAHC

FJ541745 CTRPNNNTRK SVRIGPGQTF YATGDIIGDP RQAHC

FJ541746 CTRPNNNTRK SVRIGPGQTF YATGDIIGDP RQAHC

FJ541749 CTRPNNNTRK SVRIGPGQTF YATGDIIGDP RQAHC

FJ541768 CTRPNNNTRK SVRIGPGQTF YATGDIIGDP RQAHC

FJ541769 CTRPNNNTRK SVRIGPGQTF YATGDIIGDP RQAHC

FJ541770 CTRPNNNTRK SVRIGPGQTF YATGDIIGDP RQAHC

FJ541771 CTRPNNNTRK SVRIGPGQTF YATGDIIGDP RQAHC

FJ541788 CTRPNNNTRK SVRIGPGQTF YATGDIIGDP RQAHC

FJ541790 CTRPNNNTRK SVRIGPGQTF YATGDIIGDP RQAHC

FJ541720 CTRPNNNTRK SVRIGPGQTF YATGDIIGNP RQAHC

FJ541722 CTRPNNNTRK SVRIGPGQTF YATGDIIGNP RQAHC

FJ541723 CTRPNNNTRK SVRIGPGQTF YATGDIIGNP RQAHC

FJ541738 CTRPNNNTRK SVRIGPGQTF YATGDIIGNP RQAHC

FJ541739 CTRPNNNTRK SVRIGPGQTF YATGDIIGNP RQAHC

FJ541773 CTRPNNNTRK SVRIGPGQTF YATGDIIGNP RQAHC

FJ541775 CTRPNNNTRK SVRIGPGQTF YATGDIIGNP RQAHC

FJ541781 CTRPNNNTRK SVRIGPGQTF YATGDIIGNP RQAHC

FJ541782 CTRPNNNTRK SVRIGPGQTF YATGDIIGNP RQAHC

FJ541737 CTRPNNNTRK SVRIGPGQTF YATGDLIGNP RQAHC

FJ541730 CTRPNNNTRT SVRIGPGQTF YATGDIIGNP RQAHC

FJ541733 CTRPNNNTRT SVRIGPGQTF YATGDIIGNP RQAHC

FJ541734 CTRPNNNTRT SVRIGPGQTF YATGDIIGNP RQAHC

FJ541772 CTRPNNNTRT SVRIGPGQTF YATGDIIGNP RQAHC

FJ541732 CTGPNNNTRT SVRIGPGQTF YATGDIIGNP RQAHC

FJ541740 CTKPNNNTRK SVRIGPGQTF YATGDIIGNP RQAHC

AF250251 CTRPNNNTRK STRIGPGQTF YATGDIIGNI RQAHC

U07102 CTRPNNNTRK STRIGPGQTF YATGDIIGDI RQAHC

U07101 CTRPNNNTRK SLRIGPGQTF YATGDIIGDI RRAHC

AY209194 CTRPNNNTRK SIRIGPGQTF YATGDIIGNI REAHC

AY567497 CTRPNNNTRK SIRIGPGQTF YATGDIIGNI REAHC

AY567524 CTRPNNNTRK SIRIGPGQTF YATGDIIGNI REAHC

EF117266 CTRPNNNTRK SIRIGPGQTF YATGDIIGNI REAHC

EU781878 CTRPNNNTRK SIRIGPGQTF YATGDIIGNI REAHC

EU781879 CTRPNNNTRK SIRIGPGQTF YATGDIIGNI REAHC

AF148240 CTRPNNNTRK SIRIGPGQTF YATGDIIGNI REAYC

AJ292012 CIRPNNNTRK SVRIGPGQTF YATGDIIGNI REAHC

DQ325328 CARPNNNTRK SVRIGPGQTF YATGDIIGNI RDAHC

DQ381972 CARPNNNTRK SIRIGPGQTF YATGDIIGNI REAHC

AY215067 CTRPNNNTRK SIRIGPGQTF YATGEIIGNI REAHC

EF117273 CTRPNNNTRK SIRIGPGQTF YATGAIIGNI REAHC

D13423 CTRPNNNTRK SIRIGPGQTF YATGDIIGNI RLAHC

EU781884 CTRPNNNTRK SIRIGPGQTF YATGDIIGNI RAAHC

FJ541832 CTRPNNNTRK SIRIGPGQTF YATGDIIGNI RKAHC

EU622004 CTRPNNNTRK SIRIGPGQVF YATGDIIGDI REAHC

EU622009 CTRPNNNTRK SIRIGPGQVF YATGDIIGDI REAHC

EU622010 CTRPNNNTRK SIRIGPGQVF YATGDIIGDI REAHC

EU760891 CTRPNNNTRK SIRIGPGQVF YATGDIIGDI REAHC

EU622011 CTRPNNNTRK SIRIGPGQVF YTTGDIIGDI REAHC

FJ541662 CTRPNNNTRK SVRIGPGQVF YATGDIIGDI REAHC

FJ541663 CTRPNNNTRK SVRIGPGQVF YATGDIIGDI REAHC

FJ541664 CTRPNNNTRK SVRIGPGQVF YATGDIIGDI REAHC

FJ541665 CTRPNNNTRK SVRIGPGQVF YATGDIIGDI REAHC

FJ541641 CTRPNNNTRK SVRIGPGQVF YATGDIIGNI REAHC

FJ541642 CTRPNNNTRK SVRIGPGQVF YATGDIIGNI REAHC

FJ541643 CTRPNNNTRK SVRIGPGQVF YATGDIIGNI REAHC

FJ541644 CTRPNNNTRK SVRIGPGQVF YATGDIIGNI REAHC

FJ541645 CTRPNNNTRK SVRIGPGQVF YATGDIIGNI REAHC

FJ541646 CTRPNNNTRK SVRIGPGQVF YATGDIIGNI REAHC

FJ541647 CTRPNNNTRK SVRIGPGQVF YATGDIIGNI REAHC

FJ541648 CTRPNNNTRK SVRIGPGQVF YATGDIIGNI REAHC

FJ541649 CTRPNNNTRK SVRIGPGQVF YATGDIIGNI REAHC

FJ541650 CTRPNNNTRK SVRIGPGQVF YATGDIIGNI REAHC

FJ541651 CTRPNNNTRK SVRIGPGQVF YATGDIIGNI REAHC

FJ541652 CTRPNNNTRK SVRIGPGQVF YATGDIIGNI REAHC

FJ541653 CTRPNNNTRK SVRIGPGQVF YATGDIIGNI REAHC

FJ541654 CTRPNNNTRK SVRIGPGQVF YATGDIIGNI REAHC

FJ541655 CTRPNNNTRK SVRIGPGQVF YATGDIIGNI REAHC

FJ541656 CTRPNNNTRK SVRIGPGQVF YATGDIIGNI REAHC

FJ541657 CTRPNNNTRK SVRIGPGQVF YATGDIIGNI REAHC

FJ541658 CTRPNNNTRK SVRIGPGQVF YATGDIIGNI REAHC

FJ541659 CTRPNNNTRK SVRIGPGQVF YATGDIIGNI REAHC

FJ541660 CTRPNNNTRK SVRIGPGQVF YATGDIIGNI REAHC

FJ541661 CTRPNNNTRK SVRIGPGQVF YATGDIIGNI REAHC

FJ541666 CTRPNNNTRK SVRIGPGQVF YATGDIIGNI REAHC

FJ541667 CTRPNNNTRK SVRIGPGQVF YATGDIIGNI REAHC

FJ541668 CTRPNNNTRK SVRIGPGQVF YATGDIIGNI REAHC

FJ541669 CTRPNNNTRK SVRIGPGQVF YATGDIIGNI REAHC

FJ541670 CTRPNNNTRK SVRIGPGQVF YATGDIIGNI REAHC

FJ541671 CTRPNNNTRK SVRIGPGQVF YATGDIIGNI REAHC

FJ541672 CTRPNNNTRK SVRIGPGQVF YATGDIIGNI REAHC

FJ541674 CTRPNNNTRK SVRIGPGQVF YATGDIIGNI REAHC

FJ541676 CTRPNNNTRK SVRIGPGQVF YATGDIIGNI REAHC

FJ541677 CTRPNNNTRK SVRIGPGQVF YATGDIIGNI REAHC

FJ541678 CTRPNNNTRK SVRIGPGQVF YATGDIIGNI REAHC

FJ541679 CTRPNNNTRK SVRIGPGQVF YATGDIIGNI REAHC

FJ541680 CTRPNNNTRK SVRIGPGQVF YATGDIIGNI REAHC

FJ541681 CTRPNNNTRK SVRIGPGQVF YATGDIIGNI REAHC

FJ541682 CTRPNNNTRK SVRIGPGQVF YATGDIIGNI REAHC

FJ541683 CTRPNNNTRK SVRIGPGQVF YATGDIIGNI REAHC

FJ541684 CTRPNNNTRK SVRIGPGQVF YATGDIIGNI REAHC

FJ541685 CTRPNNNTRK SVRIGPGQVF YATGDIIGNI REAHC

FJ541688 CTRPNNNTRK SVRIGPGQVF YATGDIIGNI REAHC

FJ541698 CTRPNNNTRK SVRIGPGQVF YATGDIIGNI REAHC

FJ541699 CTRPNNNTRK SVRIGPGQVF YATGDIIGNI REAHC

FJ541700 CTRPNNNTRK SVRIGPGQVF YATGDIIGNI REAHC

FJ541701 CTRPNNNTRK SVRIGPGQVF YATGDIIGNI REAHC

FJ541702 CTRPNNNTRK SVRIGPGQVF YATGDIIGNI REAHC

FJ541703 CTRPNNNTRK SVRIGPGQVF YATGDIIGNI REAHC

FJ541705 CTRPNNNTRK SVRIGPGQVF YATGDIIGNI REAHC

FJ541706 CTRPNNNTRK SVRIGPGQVF YATGDIIGNI REAHC

FJ541707 CTRPNNNTRK SVRIGPGQVF YATGDIIGNI REAHC

FJ541710 CTRPNNNTRK SVRIGPGQVF YATGDIIGNI REAHC

FJ541711 CTRPNNNTRK SVRIGPGQVF YATGDIIGNI REAHC

FJ541712 CTRPNNNTRK SVRIGPGQVF YATGDIIGNI REAHC

FJ541713 CTRPNNNTRK SVRIGPGQVF YATGDIIGNI REAHC

FJ541714 CTRPNNNTRK SVRIGPGQVF YATGDIIGNI REAHC

FJ541715 CTRPNNNTRK SVRIGPGQVF YATGDIIGNI REAHC

FJ541696 CTRPNSNTRK SVRIGPGQVF YATGDIIGNI REAHC

FJ541704 CTRSNNNTRK SVRIGPGQVF YATGDIIGNI REAHC

FJ541675 CTRPNNNTRK SVRIGPGQVS YATGDIIGNI REAHC

FJ541708 CTRPNNNTRK SVRIGPGQVF DATGDIIGNI REAHC

FJ541697 CTRPSNNTRK SVRIGPGQVF YATGDIIGNI REAHC

FJ541673 CTRPNNNTRK SARIGPGQVF YATGDIIGNI REAHC

FJ541709 CTRPNNNTRK SVRIGPGQVF YATGDIIGNI RGAHC

FJ541686 CTRPNNNTRK SIRIGPGQVF YATGDIIGNI REAHC

FJ541687 CTRPNNNTRK SIRIGPGQVF YATGDIIGNI REAHC

FJ541689 CTRPNNNTRK SIRIGPGQVF YATGDIIGNI REAHC

FJ541691 CTRPNNNTRK SIRIGPGQVF YATGDIIGNI REAHC

FJ541693 CTRPNNNTRK SIRIGPGQVF YATGDIIGNI REAHC

FJ541694 CTRPNNNTRK SIRIGPGQVF YATGDIIGNI REAHC

FJ541695 CTRPNNNTRK SIRIGPGQVF YATGDIIGNI REAHC

FJ541690 CTRPNNNTRK SIRIGPGQVF HATGDIIGNI REAHC

FJ541692 CTRPNNNTRK SIRIGPGQVF YATGEIIGNI REAHC

AF286232 CTRPNNNTRK SIRIGPGQTF YATGDIIGDI REAHC

AY567507 CTRPNNNTRK SIRIGPGQTF YATGDIIGDI REAHC

AY669748 CTRPNNNTRK SIRIGPGQTF YATGDIIGDI REAHC

DQ325331 CTRPNNNTRK SIRIGPGQTF YATGDIIGDI REAHC

DQ381973 CTRPNNNTRK SIRIGPGQTF YATGDIIGDI REAHC

EU781873 CTRPNNNTRK SIRIGPGQTF YATGDIIGDI REAHC

GU057986 CTRPNNNTRK SIRIGPGQTF YATGDIIGDI REAHC

U29179 CTRPNNNTRK SIRIGPGQTF YATGDIIGDI REAHC

AF250249 CIRPNNNTRK SIRIGPGQTF YATGTVIGDI RKAHC

AY525871 CIRPNNNTRK SIRIGPGQTF YATGDIIGDI RKAHC

EU521729 CIRPNNYTRK GIRIGPGRTV YAARKIIGDI RKVHC

EU622014 CIRPNNYTRK GIRIGPGRTV YAARKIIGDI RKVHC

EU760892 CIRPNNYTRK GIRIGPGRTV YAARKIIGDI RKVHC

EU492868 CTRPTNNTRK SIRIGPGRTF YAA-DIIGDI RLAYC

AY525879 CTRPNNNTRK SIRIGPGQTF YATGGIIGDI REAHC

U53284 CTRPNNNTRK SIRIGPGQTF YATGGIIGDI REAHC

DQ367244 CTRPNNNTRK SIRIGPGQTF YATGGIIGDI RKAHC

AY525919 CTRPNNNTRK SIRIGPGQTF YATGEIIGDI RKAHC

FJ541793 CTRPNNNTRT SIRIGPGQTF YATGDIIGDI RKAHC

FJ541802 CTRPNNNTRT SIRIGSGQTF YATGDIIGDI RKAHC

FJ541797 CTRPNNNTRT SIRIGPGQTF YATGDIIGDI RKAHC

FJ541799 CTRPNNNTRT SIRIGPGQTF YATGDIIGDI RKAHC

FJ541801 CTRPNNNTRT SIRIGPGQTF YATGDIIGDI RKAHC

FJ541803 CTRPNNNTRT SIRIGPGQTF YATGDIIGDI RKAHC

FJ541805 CTRPNNNTRT SIRIGPGQTF YATGDIIGDI RKAHC

FJ541806 CTRPNNNTRT SIRIGPGQTF YATGDIIGDI RKAHC

FJ541813 CTRPNNNTRT SIRIGPGQTF YATGDIIGDI RKAHC

FJ541821 CTRPNNNTRT SIRIGPGQTF YATGDIIGDI RKAHC

FJ541822 CTRPNNNTRT SIRIGPGQTF YATGDIIGDI RKAHC

FJ541827 CTRPNNNTRT SIRIGPGQTF YATGDIIGDI RKAHC

FJ541828 CTRPNNNTRT SIRIGPGQTF YATGDIIGDI RKAHC

FJ541837 CTRPNNNTRT SIRIGPGQTF YATGDIIGDI RKAHC

FJ541838 CTRPNNNTRT SIRIGPGQTF YATGDIIGDI RKAHC

FJ541841 CTRPNNNTRT SIRIGPGQTF YATGDIIGDI RKAHC

FJ541842 CTRPNNNTRT SIRIGPGQTF YATGDIIGDI RKAHC

FJ541844 CTRPNNNTRT SIRIGPGQTF YATGDIIGDI RKAHC

FJ541845 CTRPNNNTRT SIRIGPGQTF YATGDIIGDI RKAHC

FJ541846 CTRPNNNTRT SIRIGPGQTF YATGDIIGDI RKAHC

FJ541848 CTRPNNNTRT SIRIGPGQTF YATGDIIGDI RKAHC

FJ541851 CTRPNNNTRT SIRIGPGQTF YATGDIIGDI RKAHC

FJ541852 CTRPNNNTRT SIRIGPGQTF YATGDIIGDI RKAHC

FJ541853 CTRPNNNTRT SIRIGPGQTF YATGDIIGDI RKAHC

FJ541818 CTRPNNNTRT SIRIGPGQTF YSTGDIIGDI RKAHC

FJ541820 CTRPNNNTRT SIRIGPGQTF YSTGDIIGDI RKAHC

FJ541824 CTRPNNNTRT SIRIGPGQTF YSTGDIIGDI RKAHC

FJ541829 CTRPNNNTRT GIRIGPGQTF YATGDIIGDI RKAHC

FJ541804 CTRPNNNIRT SIRIGPGQTF YATGDIIGDI RKAHC

FJ541794 CTRPNNNTRT SIRIGPGQTF YATGDIIGDI REAHC

FJ541795 CTRPNNNTRT SIRIGPGQTF YATGDIIGDI REAHC

FJ541811 CTRPNNNTRT SIRIGPGQTF YATGDIIGDI REAHC

FJ541815 CTRPNNNTRT SIRIGPGQTF YATGDIIGDI REAHC

FJ541847 CTRPNNNTRT SIRIGPGQTF YATGDIIGDI REAHC

FJ541849 CTRPNNNTRT SIRIGPGQTF YATGDIIGDI REAHC

FJ541798 CARPNNNTRT SIRIGPGQTF YATGDIIGDI RKAHC

FJ541800 CARPNNNTRT SIRIGPGQTF YATGDIIGDI RKAHC

FJ541816 CARPNNNTRT SIRIGPGQTF YATGDIIGDI RKAHC

FJ541823 CARPNNNTRT SIRIGPGQTF YATGDIIGDI RKAHC

FJ541825 CARPNNNTRT SIRIGPGQTF YATGDIIGDI RKAHC

FJ541830 CARPNNNTRT SIRIGPGQTF YATGDIIGDI RKAHC

FJ541812 CARPNNNTRT SIRIGPGQTF YATGDIIGDI REAHC

FJ541814 CARPNNNTRT SIRIGPGQTF YATGDIIGDI REAHC

FJ541826 CTRPNNNTRT SIRIGPGQTF YATGDIIGDI RRAHC

FJ541831 CTRPNNNTRT SIRIGPGQTF YATGDIIGDI RRAHC

FJ541839 CTRPNNNTRT SIRIGPGQTF YATGDIIGDI RRAHC

DQ367253 CTRPNNNTRR SIRIGPGQTF YATGDIIGDI RKAHC

FJ541792 CTRPNNNTRK SIRIGPGQTF YATGDIIGDI RKAHC

FJ541840 CTRPNNNTRK SIRIGPGQTF YATGDIIGDI RKAHC

FJ541854 CTRPNNNTRK SIRIGPGQTF YATGDIIGDI RKAHC

FJ541860 CTRPNNNTRK SIRIGPGQTF YATGDIIGDI RKAHC

FJ541861 CTRPNNNTRK SIRIGPGQTF YATGDIIGDI RKAHC

FJ541862 CTRPNNNTRK SIRIGPGQTF YATGDIIGDI RKAHC

FJ541863 CTRPNNNTRK SIRIGPGQTF YATGDIIGDI RKAHC

FJ541864 CTRPNNNTRK SIRIGPGQTF YATGDIIGDI RKAHC

EU622006 CTRPNNNTRK SIRIGPGQTF YATGDIIGDI RLAHC

EU622007 CTRPNNNTRK SIRIGPGQTF YATGDIIGDI RLAHC

EU622008 CTRPNNNTRK SIRIGPGQTF YATGDIIGDI RLAHC

AY064240 CTRPNNNTRK SIRIGPGQTF YATGDIIGDI RAAHC

EU781869 CTRPNNNTRK SIRIGPGQTF YATGDIIGDI RAAHC

X65639 CTRPNNNTRK SIRIGPGQTF YATGDIIGDI RQAHC

X65640 CTRPNNNTRK SIRIGPGQTF YATGDIIGDI RQAHC

A37289 CTRPNNNTRK SIRIGPGQTF YATGDIIGDI RQAHC

U53302 CTRPNNNTRK SIRIGPGQTF YATGDIIGDI RQAHC

U53301 CTRPNNNTRK SIRIGPGQTF YATGDIIGDI RQAHC

AB023804 CTRPNNNTRK SIRIGPGQTF YATGDIIGDI RQAHC

A37293 CTRPNNNTRK SIRIGPGQTF YATGDIIGDI RQAHC

U53293 CTRPNNNTRK SIRIGPGQTF YATGDIIGDI RQAHC

U53292 CTRPNNNTRK SIRIGPGQTF YATGDIIGDI RQAHC

U53289 CTRPNNNTRK SIRIGPGQTF YATGDIIGDI RQAHC

U53285 CTRPNNNTRK SIRIGPGQTF YATGDIIGDI RQAHC

U53279 CTRPNNNTRK SIRIGPGQTF YATGDIIGDI RQAHC

U31363 CTRPNNNTRK SIRIGPGQTF YATGDIIGDI RQAHC

U31362 CTRPNNNTRK SIRIGPGQTF YATGDIIGDI RQAHC

U29695 CTRPNNNTRK SIRIGPGQTF YATGDIIGDI RQAHC

L07655 CTRPNNNTRK SIRIGPGQTF YATGDIIGDI RQAHC

L07654 CTRPNNNTRK SIRIGPGQTF YATGDIIGDI RQAHC

FJ940736 CTRPNNNTRK SIRIGPGQTF YATGDIIGDI RQAHC

AF148242 CTRPNNNTRK SIRIGPGQTF YATGDIIGDI RQAHC

AF148254 CTRPNNNTRK SIRIGPGQTF YATGDIIGDI RQAHC

AF101123 CTRPNNNTRK SIRIGPGQTF YATGDIIGDI RQAHC

AF101117 CTRPNNNTRK SIRIGPGQTF YATGDIIGDI RQAHC

AF101115 CTRPNNNTRK SIRIGPGQTF YATGDIIGDI RQAHC

AF101114 CTRPNNNTRK SIRIGPGQTF YATGDIIGDI RQAHC

AF148258 CTRPNNNTRK SIRIGPGQTF YATGDIIGDI RQAHC

AF148235 CTRPNNNTRK SIRIGPGQTF YATGDIIGDI RQAHC

AF250241 CTRPNNNTRK SIRIGPGQTF YATGDIIGDI RQAHC

AF392587 CTRPNNNTRK SIRIGPGQTF YATEDIIGDI RQAHC

AY525890 CTRPNNNTRK SIRIGPGQTF YAT-DIIGDI RQAHC

AF392610 CTRPNNNTRK SIRIGPGQTF YAT-DIIGDI RQAHC

AJ278382 CTRPNNNTRK SIRIGPGQTF YATDDIIGDI RQAHC

AY567523 CTRPNNNTRK SIRIGPGQTF YAT-DIIGDI RQAHC

AF392609 CTRPNNNTRK SIRIRPGQTF YAT-DIIGDI RQAHC

AF250244 CTRPNNNTRK SIRIGPGQTF YATGDIIGDI RQAHC

AF392579 CTRPNNNTRK SIRIGPGQTF YATGDIIGDI RQAHC

AF392605 CTRPNNNTRK SIRIGPGQTF YATGDIIGDI RQAHC

AJ278379 CTRPNNNTRK SIRIGPGQTF YATGDIIGDI RQAHC

AJ310094 CTRPNNNTRK SIRIGPGQTF YATGDIIGDI RQAHC

AY525875 CTRPNNNTRK SIRIGPGQTF YATGDIIGDI RQAHC

AY525876 CTRPNNNTRK SIRIGPGQTF YATGDIIGDI RQAHC

AY525877 CTRPNNNTRK SIRIGPGQTF YATGDIIGDI RQAHC

AY525933 CTRPNNNTRK SIRIGPGQTF YATGDIIGDI RQAHC

AY525944 CTRPNNNTRK SIRIGPGQTF YATGDIIGDI RQAHC

AY525946 CTRPNNNTRK SIRIGPGQTF YATGDIIGDI RQAHC

AY567496 CTRPNNNTRK SIRIGPGQTF YATGDIIGDI RQAHC

AY567513 CTRPNNNTRK SIRIGPGQTF YATGDIIGDI RQAHC

AY567519 CTRPNNNTRK SIRIGPGQTF YATGDIIGDI RQAHC

AY567537 CTRPNNNTRK SIRIGPGQTF YATGDIIGDI RQAHC

AY653049 CTRPNNNTRK SIRIGPGQTF YATGDIIGDI RQAHC

AY653066 CTRPNNNTRK SIRIGPGQTF YATGDIIGDI RQAHC

AY653067 CTRPNNNTRK SIRIGPGQTF YATGDIIGDI RQAHC

AY669738 CTRPNNNTRK SIRIGPGQTF YATGDIIGDI RQAHC

AY669742 CTRPNNNTRK SIRIGPGQTF YATGDIIGDI RQAHC

AY669744 CTRPNNNTRK SIRIGPGQTF YATGDIIGDI RQAHC

AY713414 CTRPNNNTRK SIRIGPGQTF YATGDIIGDI RQAHC

D13422 CTRPNNNTRK SIRIGPGQTF YATGDIIGDI RQAHC

D13424 CTRPNNNTRK SIRIGPGQTF YATGDIIGDI RQAHC

D13427 CTRPNNNTRK SIRIGPGQTF YATGDIIGDI RQAHC

DQ325300 CTRPNNNTRK SIRIGPGQTF YATGDIIGDI RQAHC

DQ325319 CTRPNNNTRK SIRIGPGQTF YATGDIIGDI RQAHC

DQ325323 CTRPNNNTRK SIRIGPGQTF YATGDIIGDI RQAHC

DQ325324 CTRPNNNTRK SIRIGPGQTF YATGDIIGDI RQAHC

DQ367245 CTRPNNNTRK SIRIGPGQTF YATGDIIGDI RQAHC

DQ367258 CTRPNNNTRK SIRIGPGQTF YATGDIIGDI RQAHC

DQ381980 CTRPNNNTRK SIRIGPGQTF YATGDIIGDI RQAHC

EF694032 CTRPNNNTRK SIRIGPGQTF YATGDIIGDI RQAHC

EU526663 CTRPNNNTRK SIRIGPGQTF YATGDIIGDI RQAHC

EU622001 CTRPNNNTRK SIRIGPGQTF YATGDIIGDI RQAHC

EU622002 CTRPNNNTRK SIRIGPGQTF YATGDIIGDI RQAHC

EU622015 CTRPNNNTRK SIRIGPGQTF YATGDIIGDI RQAHC

EU760888 CTRPNNNTRK SIRIGPGQTF YATGDIIGDI RQAHC

EU781871 CTRPNNNTRK SIRIGPGQTF YATGDIIGDI RQAHC

DQ367250 CTRPNNNTRK SIRIGPGQTF YATGDIIGDI RQAHC

DQ367252 CTRPNNNTRK SIRIGPGQTF YATGDIIGDI RQAHC

AF148228 CTRPNNNTRK SIRIGPGQTF YATGDIIGDI RQAHC

AF148229 CTRPNNNTRK SIRIGPGQTF YATGDIIGDI RQAHC

AF148230 CTRPNNNTRK SIRIGPGQTF YATGDIIGDI RQAHC

AF392580 CTRPNNNTRK SIRIGPGQTF YATGDIIGDI RQAHC

AF392574 CTRPNNNTRK SIRIGPGQTF YATGDIIGDI RQAHC

AJ278378 CTRPNNNTRK SIRTGPGQTF YATGDIIGDI RQAHC

AF148255 CTRPNNNTRK SIRIGPGQTF YSTGDIIGDI RQAHC

EU521727 CTRPNNNTRK SIRIGPGQTL YATGDIIGDI RQAHC

DQ325304 CTRPNNNTRK SIRIGPGQTF YATGDIIGDI RQTHC

DQ381976 CTRPNNNTRK SIRIGPGKTF YATGDIIGDI RQAHC

AY567495 CTRPNNNTRK SIRIGPGQTF YAAGDIIGDI RQAHC

AY128270 CTRPNNNTRK SIRIGPGQTF YATGDITGDI RQAHC

AY525902 CTRPNNNTRK SIRIGPGQTF YATGAITGDI RQAHC

AF067158 CTRPNNNTRK SIRIGPGQTF YATGDIMGDI RQAHC

AF067155 CTRPDNNTRK SIRIGPGQTF YATGDIIGDI RQAHC

EU908221 CTRPDNNTRK SIRIGPGQTF YATGDIIGDI RQAHC

AJ276222 CTRPHNNTRK SIRIGPGQTF YATGDIIGDI RQAHC

AJ291692 CTRPHNNTRK SIRIGPGQTF YATGDIIGDI RQAHC

AJ310091 CTRPHNNTRK SIRIGPGQTF YATGDIIGDI RQAHC

AY653051 CTRPHNNTRK SIRIGPGQTF YATGDIIGDI RQAHC

DQ325330 CTRPHNNTRK SIRIGPGQTF YATGDIIGDI RQAHC

DQ325335 CTRPHNNTRK SIRIGPGQTF YATGDIIGDI RQAHC

AF148245 CTRPSNNTRK SIRIGPGQTF YATGDIIGDI RQAHC

AF250247 CTRPSNNTRK SIRIGPGQTF YATGDIIGDI RQAHC

AY064243 CTRPSNNTRK SIRIGPGQTF YATGDIIGDI RQAHC

DQ367248 CTRPSNNTRK SIRIGPGQTF YATGDIIGDI RQAHC

DQ367257 CTRPSNNTRK SIRIGPGQTF YATGDIIGDI RQAHC

EF373536 CTRPSNNTRK SIRIGPGQTF YATGDIIGDI RQAHC

U29696 CTRPSNNTRK SIRIGPGQTF YATGDIIGDI RQAHC

U53300 CTRPSNNTRK SIRIGPGQTF YATGDIIGDI RQAHC

AY525934 CTRPSNNRRK SIRIGPGQTF YATGDIIGDI RQASC

AY567522 CTRPSNNTRK SIRIGPGQTF YATGDIIGDI RQARC

AF250255 CTRPGNNTRK SIRIGPGQTF YATGAVTGDI RQAHC

AY567515 CTRPGNNTRK SIRIGPGQTF YATGDITGDI RQAHC

AY653057 CSRPGNNTRK SIRIGPGQTF YATGDIVGDI RQAHC

DQ325334 CSRPGNNTRK SIRIGPGQTF YATGDIVGDI RQAHC

AF392602 CTRPGNNTRR SIRIGPGQTF YAAGGIIGDI RQAHC

AF392603 CTRPGNNTRK SIRIGPGQTF YAAGGIIGDI RQAHC

FJ541742 CTRPGNNTRR SVRIGPGQTF YAEGGIIGDI REAHC

FJ541758 CTRPGNNTSK SVRIGPGQTF YAEGGIIGDI REAHC

AJ276221 CTRPGNNTRQ GIRIGPGQTF YATGEIIGFI RKAHC

AJ292011 CPRPGNNTRQ GIRIGPGQTF YATGEIIGDI RKAHC

AJ278381 CTRPGNNTRQ SIRIGPGQTF YATGEIIGDI RKAHC

FJ541865 CTRPGNNTRQ SIRIGPGQTF YATGAIIGDI RQAHC

FJ541866 CTRPGNNTRQ SIRIGPGQTF YATGAIIGDI RQAHC

FJ541870 CTRPGNNTRQ SIRIGPGQTF YATGAIIGDI RQAHC

FJ541873 CTRPGNNTRQ SIRIGPGQTF YATGAIIGDI RQAHC

FJ541892 CTRPGNNTRQ SIRIGPGQTF YATGAIIGDI RQAHC

FJ541900 CTRPGNNTRQ SIRIGPGQTF YATGAIIGDI RQAHC

FJ541901 CTRPGNNTRQ SIRIGPGQTF YATGAIIGDI RQAHC

FJ541903 CTRPGNNTRQ SIRIGPGQTF YATGAIIGDI RQAHC

FJ541907 CTRPGNNTRQ SIRIGPGQTF YATGAIIGDI RQAHC

FJ541908 CTRPGNNTRQ SIRIGPGQTF YATGAIIGDI RQAHC

FJ541913 CTRPGNNTRQ SIRIGPGQTF YATGAIIGDI RQAHC

FJ541914 CTRPGNNTRQ SIRIGPGQTF YATGAIIGDI RQAHC

FJ541917 CTRPGNNTRQ SIRIGPGQTF YATGAIIGDI RQAHC

FJ541921 CTRPGNNTRQ SIRIGPGQTF YATGAIIGDI RQAHC

FJ541874 CTRPGNNTRQ SIRIGPGQTF YSTGAIIGDI RQAHC

FJ541887 CTRPGNNTRQ SIRIGPGQTF YATGAKIGDI RQAHC

FJ541867 CTRPGNNARQ SIRIGPGQTF YATGAIIGDI RQAHC

FJ541868 CTRPGNNTRQ SIRIGPGQTF YATGAIIGDI RQAYC

FJ541876 CTRPGNNTRQ SIRIGPGQTF YATGAIIGDI RQAYC

FJ541884 CTRPGNNTRQ SIRIGPGQTF YATGAIIGDI RQAYC

FJ541888 CTRPGNNTRQ SIRIGPGQTF YATGAIIGDI RQAYC

FJ541891 CTRPGNNTRQ SIRIGPGQTF YATGAIIGDI RQAYC

FJ541893 CTRPGNNTRQ SIRIGPGQTF YATGAIIGDI RQAYC

FJ541894 CTRPGNNTRQ SIRIGPGQTF YATGAIIGDI RQAYC

FJ541895 CTRPGNNTRQ SIRIGPGQTF YATGAIIGDI RQAYC

FJ541896 CTRPGNNTRQ SIRIGPGQTF YATGAIIGDI RQAYC

FJ541897 CTRPGNNTRQ SIRIGPGQTF YATGAIIGDI RQAYC

FJ541898 CTRPGNNTRQ SIRIGPGQTF YATGAIIGDI RQAYC

FJ541899 CTRPGNNTRQ SIRIGPGQTF YATGAIIGDI RQAYC

FJ541902 CTRPGNNTRQ SIRIGPGQTF YATGAIIGDI RQAYC

FJ541904 CTRPGNNTRQ SIRIGPGQTF YATGAIIGDI RQAYC

FJ541906 CTRPGNNTRQ SIRIGPGQTF YATGAIIGDI RQAYC

FJ541909 CTRPGNNTRQ SIRIGPGQTF YATGAIIGDI RQAYC

FJ541910 CTRPGNNTRQ SIRIGPGQTF YATGAIIGDI RQAYC

FJ541911 CTRPGNNTRQ SIRIGPGQTF YATGAIIGDI RQAYC

FJ541912 CTRPGNNTRQ SIRIGPGQTF YATGAIIGDI RQAYC

FJ541915 CTRPGNNTRQ SIRIGPGQTF YATGAIIGDI RQAYC

FJ541918 CTRPGNNTRQ SIRIGPGQTF YATGAIIGDI RQAYC

FJ541919 CTRPGNNTRQ SIRIGPGQTF YATGAIIGDI RQAYC

FJ541920 CTRPGNNTRQ SIRIGPGQTF YATGAIIGDI RQAYC

FJ541922 CTRPGNNTRQ SIRIGPGQTF YATGAIIGDI RQAYC

FJ541923 CTRPGNNTRQ SIRIGPGQTF YATGAIIGDI RQAYC

FJ541924 CTRPGNNTRQ SIRIGPGQTF YATGAIIGDI RQAYC

FJ541925 CTRPGNNTRQ SIRIGPGQTF YATGAIIGDI RQAYC

FJ541927 CTRPGNNTRQ SIRIGPGQTF YATGAIIGDI RQAYC

FJ541929 CTRPGNNTRQ SIRIGPGQTF YATGAIIGDI RQAYC

FJ541931 CTRPGNNTRQ SIRIGPGQTF YATGAIIGDI RQAYC

FJ541932 CTRPGNNTRQ SIRIGPGQTF YATGAIIGDI RQAYC

FJ541926 CTRPGNNTRQ SIRIGPGQTF YATGAIIGDI RQTYC

FJ541905 CTRPGNNTRQ SIRIGPGQTS YATGAIIGDI RQAYC

FJ541877 CIRPGNNTRQ SIRIGPGQTF YATGAIIGDI RQAYC

FJ541878 CIRPGNNTRQ SIRIGPGQTF YATGAIIGDI RQAYC

FJ541916 CIRPGNNTRQ SIRIGPGQTF YATGAIIGDI RQAHC

FJ541930 CIRPGSNTRQ SIRIGPGQTF YATGAIIGDI RQAHC

FJ541869 CTRPGNNTRQ SIRIGPGQTF YATGDIIGDI RQAHC

FJ541871 CTRPGNNTRQ SIRIGPGQTF YATGDIIGDI RQAHC

FJ541889 CTRPGNNTRQ SIRIGPGQTF YATGDIIGDI RQAHC

FJ541890 CTRPGNNTRQ SIRIGPGQTF YATGDIIGDI RQAHC

FJ541872 CTRSGNNTRQ SIRIGPGQTF YATGDIIGDI RQAHC

FJ541875 CTRPGNNTRQ SIRIGPGQTF YATGDIIGDV RQAHC

DQ325327 CSRPGNNTRQ SIRIGPGQTF YATGDIIGDI RQAHC

AY525885 CTRPGNNTRK SIRIGPGQTF YATGDIIGDI RQAHC

DQ367254 CTRPGNNTRK SIRIGPGQTF YATGDIIGDI RQAHC

EF117269 CTRPGNNTRK SIRIGPGQTF YATGDIIGDI RQAHC

FJ541987 CTRPGNNTRK SIRIGPGQTF YATGDIIGDI RQAHC

AY567514 CTRPGNNTRK SIRIGPGQTF YATGDIIGDP RQAHC

AY653052 CTRPKNNTRK SIRIGPGQTF YAYGDIIGDI RDAHC

DQ325336 CTRPGNNTRK SIRIGPGQTF YATGDIIGDI RDAHC

AY653061 CTRPGNNTRK SIRIGPGQTF YATGDIIGDI RRAHC

EU781859 CTRPGNNTRK SIRIGPGQTF YATGDIIGDI REAHC

EU492869 CTSPTSNTRK SIRIGPGQTF YATGDIIGDI RQAHC

EU622003 CTGPNNNTRK SIRIGPGQTF YATGDIIGDI RQAHC

AF392559 CTRPNNNTRR SIRIGPGQTF YATGDIIGDI RQAHC

AF392560 CTRPNNNTRR SIRIGPGQTF YATGDIIGDI RQAHC

AF392561 CTRPNNNTRR SIRIGPGQTF YATGDIIGDI RQAHC

AF392562 CTRPNNNTRR SIRIGPGQTF YATGDIIGDI RQAHC

AF392563 CTRPNNNTRR SIRIGPGQTF YATGDIIGDI RQAHC

EU760889 CTRPNNNTRR SIRIGPGQTF YATGDIIGDI RQAHC

EU760890 CTRPNNNTRR SIRIGPGQTF YATGDIIGDI RQAHC

AF148259 CTRPNNNTRR SIRIGPGQTF YATGDVIGDI RQAHC

AY049009 CTRPNNNTRR SIRIGPGQVF YANNDIIGDI RQAHC

AY775283 CTRPNNNTRR SIRIGPGQVF YANNDIIGDI RQAHC

AY049010 CTRPNNNTRR SIRIGPGLVF YANNDIIGDI RQAHC

DQ381975 CTRPNNNTRR SIRIGPGQVF YATNDIIGDI RQAHC

AY525847 CIRPNNNTRR SIRIGPGQTF YATGDIIGDI RQAHC

AJ278377 CTRPNNNTRE SIRIGPGQTF YATGDIIRDI RSAHC

DQ367255 CTRPSNNTRE SIRIGPGQTF YATGDIIGDI REAHC

AY128269 CTRPNNNTRE SIRIGPGQTF YATGDIIGDI RQAHC

AY128271 CTRPNNNTRE SIRIGPGQTF YATGDIIGDI RQAHC

AY653059 CTRPNNNTRE SIRIGPGQTF YATGDIIGDI RQAHC

AY128262 CTRPNNNTRE SIRIGPGQTF YASGDIIGDI RQAHC

AJ291689 CTRPNNNTRE SIRIGPGQTF YATGDIIGDI RQAQC

EU492870 CTRPNNNTRD SIRIGPGQTF YATGDIIGDI RQAHC

AF392564 CIRPNNNTRK SIRIGPGQTF YATGDIIGDI RQAHC

AF392565 CIRPNNNTRK SIRIGPGQTF YATGDIIGDI RQAHC

AF392566 CIRPNNNTRK SIRIGPGQTF YATGDIIGDI RQAHC

AY128263 CIRPNNNTRK SIRIGPGQTF YATGDIIGDI RQAHC

AY567501 CIRPNNNTRK SIRIGPGQTF YATGDIIGDI RQAHC

AY653063 CIRPNNNTRK SIRIGPGQTF YATGDIIGDI RQAHC

AY653064 CIRPNNNTRK SIRIGPGQTF YATGDIIGDI RQAHC

AY653065 CIRPNNNTRK SIRIGPGQTF YATGDIIGDI RQAHC

DQ325338 CIRPNNNTRK SIRIGPGQTF YATGDIIGDI RQAHC

DQ398880 CIRPNNNTRK SIRIGPGQTF YATGDIIGDI RQAHC

DQ398881 CIRPNNNTRK SIRIGPGQTF YATGDIIGDI RQAHC

EU781882 CIRPNNNTRK SIRIGPGQTF YATGDIIGDI RQAHC

AY567535 CIRPNNKTRK SIRIGPGQTF YATGDIIGDI RQAHC

EU781857 CIRPNNNTRK SIRIGPGQTF YARGDIIGDI RQAHC

AF392608 CVRPHNNTRK SIRIGPGQTF YATEEIIGDI RQAHC

DQ325308 CIRPGNNTRK SIRIGPGQTF YATEAIIGDI RQAHC

AY567536 CIRPNNNTRK SIRIGPGQTF YGTN-IIGDI RQAHC

AY525886 CIRPNNNTRK SIRIGPGQTF YATGRIIGDI RQAHC

AY525950 CIRPNNNTRK SIRIGPGQTF YATGRIIGDI RQAHC

AY064238 CIRPGNNTRK SIRIGPGQTF YATGDIIGDI RQAFC

AY653058 CIRPGNNTRK SIRIGPGQTF YATGDIIGDI RQAPC

AY525856 CIRPGNNTRK SIRIGPGQTF YATGDIIGDI RQAHC

AY525941 CIRPGNNTRK SIRIGPGQTF YATGDIIGDI RQAHC

EU781893 CIRPGNNTRK VYRIGPGQTF YATGDIIGDI RQAHC

AY567503 CIRPSNNTRK SIRIGPGQTF YATGDITGDI RRAHC

AY567511 CIRPGNNTRK SIRIGPGQTF YATEDITGNI REAHC

EU781858 CIRPGNNTRK SIRIGPGQTF YATGDIIGDI REAHC

AY567510 CIRPGNNTRK SIRIGPGQTF YATGDIIGDV RQAHC

AY567520 CIRPSNNTRK SIRIEPGQTF YATGDIIGDV RQAHC

AY525865 CIRPNNNTRK SIRFGPGQAF FATGDIIGDI RQAQC

U29697 CARPNNNTRK SIRIGPGQAF FATGDIIGDI RQAHC

EU781875 CIRPNNNTRK SVRIGPGQMF FAT-DIIGDI RQAHC

AY525906 CIRPGNNTSK SIRIGPGQTF FATDRIIGDI RQAYC

DQ149147 CIRPGNNTRK SIRIGPGQTF FATGEIIGDI RQAHC

AY567533 CIRPGNNTRK SIRIGPGQTF FATGDIIGDI RQAHC

AF392597 CVRPNNNTRK SVRIGPGQTF YATGEIIGKI RQAYC

AF392598 CVRPNNNTRK SVRIGPGQTF YATGEIIGKI RQAYC

AF392599 CVRPNNNTRK SVRIGPGQTF YATGEIIGKI RQAYC

AY567518 CERPNNNTRK SVRIGPGQTF YATGDIIGNI RQAYC

AY567527 CIRPNNDTRK SVRIGPGQTF YATGQIIGDI RQAYC

DQ325307 CIRPNNNTRK SVRIGPGQTF YATGDIIGDI RQAYC

EF694034 CIRPNNNTRK SKRIGPGQTF YATGDIIGDI RQAYC

FJ541980 CIRPNNNTRK SIRIGPGQTF YATGEIIGNI RQAYC

FJ541981 CIRPNNNTRK SIRIGPGQTF YATGEIIGNI RQAYC

FJ541982 CIRPNNNTRK SIRIGPGQTF YATGEIIGNI RQAYC

FJ541983 CIRPNNNTRK SIRIGPGQTF YATGEIIGNI RQAYC

FJ541985 CIRPNNNTRK SIRIGPGQTF YATGEIIGNI RQAYC

FJ541984 CIRPNNNTRK SIRIGPGQTF YATGEIIGNI RLAYC

AY525868 CIRPNNNTRK SIRIGPGQTF YATGDIIGNI KQAYC

AF392606 CIRPNNNTRK SIRIGPGQTF YATGGIIGDI RQAYC

AF392607 CIRPHNNTRK SIRIGPGQTF YATGGIIGDI RQAYC

AY525915 CIRPNNNTRK SIRIGPGQTF YATGEIIGDI RQAYC

EF694035 CIRPNNNTRK SIRIGPGQTF YATGDIIGDI RQAYC

EF694036 CIRPNNNTRK SIRIGPGQTF YATGDIIGDI RQAYC

EF117272 CIRPNNNTRK SIRIGPGQTF YATGDIVGDI RQAYC

EU781853 CIRPNNNTRE SIRIGPGQTF YATGDIIGDI RQAYC

AY567528 CIRPNNNTRK SIRVGPGQTF YATGDIIGDI RSAYC

EU526662 CIRPSNNTRK SIRIGPGQTF YATGDVIGDI RQAYC

U53290 CIRPSNNTRK SIRIGPGQTF YATGDIIGDI RQAYC

AF392573 CARPNNNTRK SIRIGPGQTF YATGDIIGDI RQAYC

EU908214 CIRPNNNTRK SIRIGPGQTF YATGDIIGDI RQASC

EU908215 CIRPNNNTRK SIRIGPGQTF YATGDIIGDI RQASC

AJ292013 CARPSNNTRQ SIRIGPGQTF YATGEIIGDI RQAHC

AY567534 CIRPSNNTRQ SIRIGPGQTF YATGEIIGDI RQAHC

AY653048 CARPSNNTRK SIRIGPGQTF YATGEIIGDI RQAHC

EU521728 CARPSNNTRK SIRIGPGQTF YATGEIIGDI RQAHC

EU622005 CARPSNNTRK SIRIGPGQTF YATGEIIGDI RQAHC

DQ325322 CQRPSNNTRQ SIRIGPGQTF YATGDITGDI RQAHC

EF117271 CARPSNNTRT SIRIGPGQTF YATGAITGDI RQAHC

U53287 CARPSNNTRK SIRIGPGQTF YATGDIIGDI RQAHC

U53297 CARPSNNTRK SIRIGPGQTF YATGDIIGDI RQAHC

DQ325314 CARPANNTRK SIRIGPGQTF YATGDIIGDI RQAHC

DQ325315 CARPNNNTRK SIRIGPGQTF YATGEIIGDI REAHC

EU781876 CARPNNNTRK SIRIGPGQTF YATGDIIGDI REAHC

FJ541850 CARPNNNTRK SIRIGPGQTF YATGDIIGDI RKAHC

FJ541855 CARPNNNTRK SIRIGPGQTF YATGDIIGDI RKAHC

FJ541856 CARPNNNTRK SIRIGPGQTF YATGDIIGDI RKAHC

FJ541857 CARPNNNTRK SIRIGPGQTF YATGDIIGDI RKAHC

FJ541858 CARPNNNTRK SIRIGPGQTF YATGDIIGDI RKAHC

FJ541859 CARPNNNTRK SIRIGPGQTF YATGDIIGDI RKAHC

FJ541843 CARPNNNTRK SIRIGPGQTF YATGDIIGDI RRAHC

DQ381974 CARPNNNTRK SIRIGPGQTF YATGDIIGDI RQAHC

U29698 CARPNNNTRK SIRIGPGQTF YATGDIIGDI RQAHC

AY525903 CMRPNNNTRK SIRIGPGQTF YATGDIIGDI RQAHC

DQ149152 CKRPNNNTRK SIRIGPGQTF YATGDIIGDI RQAHC

AJ291690 CSRPNNNTRK SIRIGPGQTF YATGDIIGDI RQAHC

AJ310093 CVRPNNNTRK SIRIGPGQTF YATGDIIGDI RQAHC

DQ149139 CVRPNNNTRK SIRIGPGQTF YATGDIIGDI RQAHC

DQ367246 CVRPNNNTRK SIRIGPGQTF YATGDIIGDI RQAHC

AF148253 CTRPNNNTRK SIRIGPGQAF YATGDIIGDI RQAHC

AF250261 CTRPNNNTRK SIRIGPGQAF YAHGDIIGDI RQAHC

AJ292006 CTRPNNNTRK SIPLGPGKAW YTTGQIIGNI RQAHC

AJ292007 CIRPNNNTRK SIPLGPGKAW YTTGQIIPDI RQAHC

AY128272 CTRPVNNTRK SISLGLGRAL YTTGQIIGDI RQAHC

AF250258 CTRPNNNTRK SIPIGPGQAF YATGGIIGDI RQAHC

AF286223 CTRPNNNTRK SIRIGPGQAF YATNGIIGDI RQAHC

DQ404010 CTRPNNNTRK SIRIGPGQAF YATNGIIGDI RQAHC

AY567525 CTRPYNNTRK SIRIGPGQAF YATGEIIGNI RQAYC

AY567538 CTRPYNNTRK SIRIGPGQAF YATGEIIGNI RQAYC

EU781868 CTRPGNNTRK SIRIGPGQAF YATGEIIGKI RDAYC

AY209193 CTRPGNNTRK SIRIGPGQAF YATGEIIGNI RQAHC

DQ381978 CTRPGNNTRK SIRIGPGQAF YATGEIIGDI RQAHC

EU492871 CTRPGNNTRK SIRIGPGQAF YATGGIIGDI RRAHC

AY525896 CTRPGNNTRK SIRIGPGQAF YATEDIIGDI RQAHC

DQ325332 CTRPNNNTRK SIRIGPG-SI LCTGDIIGDI RQAHC

DQ367260 CTRPNNNTRK SIRIGPGQSF YATGDIIGNI RQAHC

DQ367247 CTRPNNNTRK SIRIGPGQMF YATGDIIGDI RQAHC

AF101120 CTRPNNNTRK SIRIGPGQTF YATGDIIGDI RRAYC

D13426 CTRPNNNTRK SIRIGPGQTF YATGDIIGDI RRAYC

S66381 CTRPNNNTRK SIRIGPGQTF YATGDIIGDI RRAYC

U07098 CTRPNNNTRK SIRIGPGQTF YATGDIIGDI RRAYC

U07103 RTRPNNNTRK SIRIGPGQTF YATGDIIGDI RRAYC

AF148248 CTRPNNNTRK SIRIGPGQTF YATGDIIGDI RLAYC

AY567517 CTRPNNNTRK SIRIGPGQTF YATGDVIGNI RAAYC

AY653045 CTRPNNNTRK SIRIGPGQTF YATGDGIGDI RAAYC

AJ311642 CTRPTNNTRK SIRIGPGQTF YATGDIIGDI RAAYC

AJ278376 CTRPNNNTRK SIRIGPGQTF YATGDIIGDI RKAYC

AY567498 CTRPNNNTRK SIRIGPGQTF YATGDIIGDI RKAYC

EU781854 CTRPNNNTRK SIRIGPGQTF YATGDIIGDI RKAYC

DQ325325 CVRPNNNTRK SIRIGPGQTF YATGEIIGDI RKAYC

EU781889 CTRPNNNTRK SIRIGPGQTF YATGEIIGDI RKAYC

AY525870 CTRPNNNRRK SIRIGPGQTF YATGAIIGDI RKAYC

AF250245 CTRPGNNTRK SIRIGPGQTF YATGDIIGDI RKAYC

AF392592 CTRPGNNARK SIRIGPG-TF YATGDIIGDI RKAYC

AF392593 CTRPGNNTRK SIRIGPGQTF YATGDIIGDI RKAYC

EU781862 CTRPGNNTRE SIRIGPGQTF YATGDIIGDI RKAYC

AY567506 CTRPGNNTRK SIRIGPGQTF YATGAIIGDI RRAYC

AJ291691 CTRPGNNTRK SIRIGPGQTF YATGDIIGDI RQAYC

FJ541879 CTRPGNNTRK SIRIGPGQTF YATGDIIGDI RQAYC

FJ541928 CTRPGNNTRK SIRIGPGQTF YATGDIIGDI RQAYC

FJ541881 CTRPGNNSRK SIRIGPGQTF YATGDIIGDI RQAYC

FJ541882 CTRPGNNSRK SIRIGPGQTF YATGDIIGDI RQAYC

FJ541883 CTRPGNNSRK SIRIGPGQTF YATGDIIGDI RQAYC

FJ541885 CTRPGNNSRK SIRIGPGQTF YATGDIIGDI RQAYC

FJ541886 CTRPGNNSRK SIRIGPGQTF YATGDIIGDI RQAYC

FJ541880 CTRPGNNSRK SIRIGPGQIF YATGDIIGDI RQAYC

AF148257 CTRPNNNTRK SIRIGPGQTF YATGEIIGDI RQAYC

AY525880 CTRPNNNTRK SIRIGPGQTF YATGEIIGDI RQAYC

AY525930 CTRPNNNTRK SIRIGPGQTF YATGEIIGDI RQAYC

AY525937 CTRPNNNTRK SIRIGPGQTF YATGEIIGDI RQAYC

AY653062 CTRPNNNTRK SIRIGPGQTF YATGEIIGDI RQAYC

D13421 CTRPNNNTRK SIRIGPGQTF YATGEIIGDI RQAYC

DQ325312 CTRPNNNTRK SIRIGPGQTF YATGEIIGDI RQAYC

AF250250 CTRPNNNTRK SIRIGPGQTF YAAGEIIGDI RQAYC

AY567504 CTRPNNNTRK SIRIGPGQTF YATGEIIGDI RHAYC

AJ292009 CTRPNNNTRK SIRIGPGQTF YATGGIIGDI RQAYC

DQ367259 CTRPNNNTRK SIRIGPGQTF YATGGVIGDI RQAYC

AF148232 CTRPNNNTRQ SIRIGPGQAF YATGEIIGDI RQAYC

AF148233 CTRPNNNTRQ SITIGPGQAF YATGDIIGDI RQAYC

EU908218 CTRPNNNTRQ SIRIGPGQVF YATGAIIGDI RQAYC

EU908219 CTRPNNNTRQ SIRIGPGQVF YATGAIIGDI RQAYC

EU908220 CTRPNNNTRQ SIRIGPGQVF YATGAIIGDI RQAYC

AF392590 CTRPNNNTRQ SIRIGPGQTF YATGDIIGDI RQAYC

AF392591 CTRPNNNTRR SIRIGPGQTF YATGDIIGDI RQAYC

AY567539 GTRPNNSTRR SIRIGPGQTF YATGAIIGDI RQAYC

AF392594 CTRPNNNKRT SIRIGPGQTF FATGQIIGNI RQAYC

AF392596 CTRPNNNRRT SIRIGPGQTF FATGEITGNI RQAYC

AF392595 CTRPNNNKRT SIRIGPGQTF FATGDIIGDI RQAYC

AY064241 CTRPNNNTRT SIRIGPGQTF YATGDIIGDI RQAYC

DQ149145 CTRPNNNTRT SIRIGPGQTF YATGDIIGDI RQAYC

EU622000 CTRPNNNTRT SVRIGPGQTF YATGDIIGDI RKAYC

EU622012 CTRPNNNTRT SVRIGPGQTF YATGDIIGDI RKAYC

EU622013 CTRPNNNTRT SVRIGPGQTF YATGDIIGDI RKAYC

AY525951 CTRPNNNKRT SVRIGPGQTF YATGDIIGDI REAYC

FJ541808 CTRPNNNTRT SIRIGPGQTF YATGDIIGDI RKAYC

FJ541809 CTRPNNNTRT SIRIGPGQTF YATGDIIGDI RKAYC

FJ541810 CTRPNNNTRT SIRIGPGQTF YATGDIIGDI RKAYC

FJ541817 CTRPNNDTRT SIRIGPGQTF YATGDIIGDI RKAYC

DQ325302 CTRPNNNIRR SIRIGPGQTF YATGDIIGDI REAYC

AF250252 CTRPNNNRRK SIRIGPGQTF YATGDIIGDI RQAYC

AF392568 CTRPNNNARK SIRIGPGQTF YATGDIIGDI RQAYC

FJ541953 CTRPNNNIRK SIRIGPGQTF YATGDIIGDI RQAYC

FJ541957 CTRPNNNIRK SIRIGPGQTF YATGDIIGDI RQAYC

FJ541958 CTRPNNNIRK SIRIGPGQTF YATGDIIGDI RQAYC

FJ541960 CTRPNNNIRK SIRIGPGQTF YATGDIIGDI RQAYC

FJ541961 CTRPNNNIRK SIRIGPGQTF YATGDIIGDI RQAYC

FJ541962 CTRPNNNIRK SIRIGPGQTF YATGDIIGDI RQAYC

FJ541966 CTRPNNNIRK SIRIGPGQTF YATGDIIGDI RQAYC

FJ541967 CTRPNNNIRK SIRIGPGQTF YATGDIIGDI RQAYC

FJ541968 CTRPNNNIRK SIRIGPGQTF YATGDIIGDI RQAYC

FJ541969 CTRPNNNIRK SIRIGPGQTF YATGDIIGDI RQAYC

FJ541970 CTRPNNNIRK SIRIGPGQTF YATGDIIGDI RQAYC

FJ541993 CTRPNNNIRK SIRIGPGQTF YATGDIIGDI RQAYC

FJ541994 CTRPNNNIRK SIRIGPGQTF YATGDIIGDI RQAYC

FJ541959 CTRSNNNIRK SIRIGPGQTF YATGDIIGDI RQAYC

AF148247 CTRPNNNTRK SIRIGPGQTF YATGDIIGDI RQAYC

AF148252 CTRPNNNTRK SIRIGPGQTF YATGDIIGDI RQAYC

AF250242 CTRPNNNTRK SIRIGPGQTF YATGDIIGDI RQAYC

AF392569 CTRPNNNTRK SIRIGPGQTF YATGDIIGDI RQAYC

AF392570 CTRPNNNTRK SIRIGPGQTF YATGDIIGDI RQAYC

AF392571 CTRPNNNTRK SIRIGPGQTF YATGDIIGDI RQAYC

AF392572 CTRPNNNTRK SIRIGPGQTF YATGDIIGDI RQAYC

AF392575 CTRPNNNTRK SIRIGPGQTF YATGDIIGDI RQAYC

AF392578 CTRPNNNTRK SIRIGPGQTF YATGDIIGDI RQAYC

AF392588 CTRPNNNTRK SIRIGPGQTF YATGDIIGDI RQAYC

AJ310092 CTRPNNNTRK SIRIGPGQTF YATGDIIGDI RQAYC

AY653050 CTRPNNNTRK SIRIGPGQTF YATGDIIGDI RQAYC

DQ149150 CTRPNNNTRK SIRIGPGQTF YATGDIIGDI RQAYC

DQ325318 CTRPNNNTRK SIRIGPGQTF YATGDIIGDI RQAYC

EF694033 CTRPNNNTRK SIRIGPGQTF YATGDIIGDI RQAYC

FJ541976 CTRPNNNTRK SIRIGPGQTF YATGDIIGDI RQAYC

FJ541991 CTRPNNNTRK SIRIGPGQTF YATGDIIGDI RQAYC

FJ541992 CTRPNNNTRK SIRIGPGQTF YATGDIIGDI RQAYC

L07651 CTRPNNNTRK SIRIGPGQTF YATGDIIGDI RQAYC

U07099 CTRPNNNTRK SIRIGPGQTF YATGDIIGDI RQAYC

U53280 CTRPNNNTRK SIRIGPGQTF YATGDIIGDI RQAYC

U53282 CTRPNNNTRK SIRIGPGQTF YATGDIIGDI RQAYC

X68406 CTRPNNNTRK SIRIGPGQTF YATGDIIGDI RQAYC

AY567532 CERPNNNTRT STRIGPGQAF YAMGDIIGDI RQASC

EU781864 CIRPNNNTRT SIHIGPGQAF YAMGDIIGDI KKAYC

AY653054 CIRPGNNTRK SVRIGPGQAF YATGDIIGDI RRAYC

DQ325306 CIRPGNNTRK SVRIGPGQAF YATGDIIGDI RRAYC

AY653055 CIRPGNNTRK SIRIGPGQAF YATGDIIGDI RRAYC

AY653056 CVRPNNNRRK SVRIGPGQAF YATGDIVGDI RRAYC

AF101126 CTRPNNNTRK SIRIGPGQAF YATGDIIGDI RQAYC

AY209192 CTRPNENRRK SIRIGPGQAF YATGDIIGDI RQARC

EF117267 CTRPNENRRK SIRIGPGQAF YATGDIIGDI RQARC

AY049708 CTRPNNNTRK SIRIGPGQMF YATGDIIGDI RQAYC

AY049709 CTRPNNNTRK SIRIGPGQMF YATGDIIGDI RQAYC

AY049710 CTRPNNNTRK SIRIGPGQMF YATGDIIGDI RQAYC

AY049711 CTRPNNNTRK SIRIGPGQMF YATGDIIGDI RQAYC

U29694 CTRPNNNTRK SIRIGPGQTF YATGDIIGDI RQRNL

AJ278374 CTRPNNNTRK SIRIGPGQTF YATGEIIGNI R-RHC

DQ325313 WTRPPNNTRK SIMMGPGRTF FAAGGIIGNI HTSTL

AY653043 CTRPNNNTRK SIRIGPGQTF YAKEEG-GEN KTSTL

AY653046 CTRPSNNTRK SIRIGPGQTF YATGDGMGRH KTSTL
