# Supplementary material for: Co-receptor tropism prediction among 1045 Indian HIV-1 subtype C sequences: Therapeutic implications for India
Source: AIDS Res Ther. 2010 Jul 21;7:24. doi: 10.1186/1742-6405-7-24 (PMC2918521; doi:10.1186/1742-6405-7-24)
Supplement: Additional file 2 — Multiple sequence analysis of Env V3 region of clinical isolates: Multiple sequence analysis was carried out in ClustalW. Dots represent residual similarity with consensus C sequences downloaded from Los Alamos Database. Dash indicates deletion in that position. [file 1742-6405-7-24-S2.DOC]

1 35

CON_C CTRPNNNTRKSIRIGPGQTFYATGDIIGDIRQAHC

SJNAHS01 ...........V.......................

SJNAHS02 ....S......V.......................

SJNAHS03 ..................V..G......E......

SJNAHS04 .....H..G.G..V........M..V.........

SJNAHS06 ......................M..V....K....

SJNAHS07 ....S......V.......................

SJNAHS09 ....G.....................T......Y.

SJNAHS10 ....G............................Y.

SJNAHS11 ...........V.......................

SJNAHS12 ....G.....................T......Y.

SJNAHS13 .A...................T..QV..N..E...

SJNAHS16 ...........V.....................Y.

SJNAHS17 ........................E..........

SJNAHS18 .I.........V...........-N...N....Y.

SJNAHS19 ...........V............N........Y.

* **.:** *.:*:****.** :: *:*::*:*

**Additional File 2**
