# Supplementary figures and images for: Co-receptor tropism prediction among 1045 Indian HIV-1 subtype C sequences: Therapeutic implications for India
Source: AIDS Res Ther. 2010 Jul 21;7:24. doi: 10.1186/1742-6405-7-24 (PMC2918521; doi:10.1186/1742-6405-7-24)

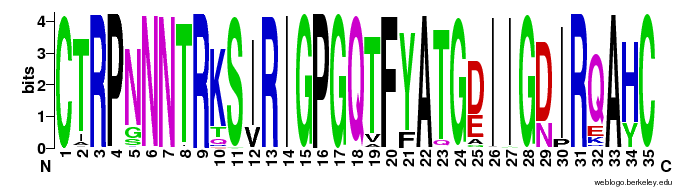


**HIV-1 subtype C strains**

**A.**

**HIV-1 subtype B strains**

**B.**

**
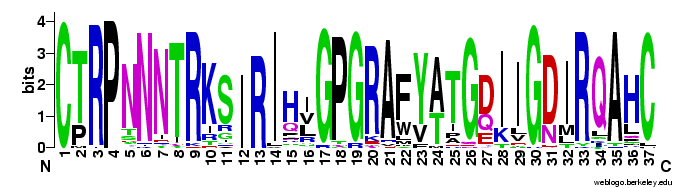
**

**C.**

**
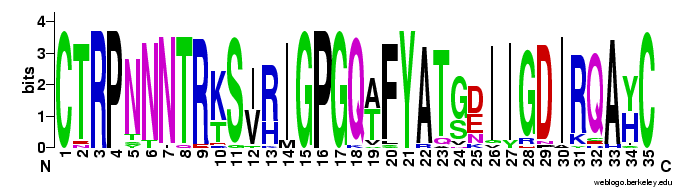
**

**HIV-1 subtype A/A1 strains**

**Additional File 3**

Supplement: Additional file 3 — Subtype specific Consensus sequence logo. Consensus sequence logos of A. Subtype C strains (n = 1045), B. Subtype B (n = 56) and C. Subtype A/A1 strains (n = 17) irrespective of the co-receptor tropism. Consensus sequence logos were created using WebLogo ver 3 http://weblogo.berkeley.edu/logo.cgi. [file 1742-6405-7-24-S3.DOC]
